# Physiological stress response to hydropeaking in rainbow trout (*Oncorhynchus mykiss*)

**DOI:** 10.1007/s10695-026-01633-z

**Published:** 2026-01-16

**Authors:** Raul Hernandez-Marchena, Álvaro De la Llave-Propín, Joaquín Solana-Gutiérrez, María Dolores Bejarano

**Affiliations:** 1 Department of Systems and Natural Resources, Universidad Politécnica de Madrid, 28040 Madrid, Spain; 2 Department of Agrarian Production, Universidad Politécnica de Madrid, 28040 Madrid, Spain

**Keywords:** Ecological restoration, Hydropower plant, Mesocosms, Pigmentation

## Abstract

In recent years, there has been growing interest in investigating hydropeaking’s impact on rivers. In the case of fish, literature documents stranding, loss of spawning beds, and behavioural changes, while the physiological stress response is less understood. In this study, a natural flow scenario and five hydropeaking scenarios were simulated in a fluvial mesocosm named Greenchannel. Scenarios were characterised through hydraulic/hydrological (water velocity and level, water level fall rate, and frequency and number of inundations) and water quality (temperature, dissolved oxygen levels, turbidity) variables. Different test groups of 15 rainbow trout (*Oncorhynchus mykiss*) (*n* = 90) were subjected to these scenarios for 24 h each. Cortisol, creatine phosphokinase (CPK), lactate dehydrogenase (LDH), triglycerides (TGC), lactate, non-esterified fatty acids (NEFA), and histological pigmentation were measured at the end of the tests. The variation in physiological variables in response to the environmental variables characterising each scenario was assessed. The levels of cortisol, lactate, TGC, a pigmentation parameter (i.e. lightness), and water turbidity varied significantly with increasing intensities of hydropeaking, mainly velocity and rate of change of water levels. The mobilisation of metabolites suggests a prolonged stress response extending beyond the acute phase as a result of hydropeaking. Developed environment-physiology models pointed to specific environmental thresholds for rainbow trout (for instance, 0.1 m·s^−1^ (velocity) and 0.13 m·h^−1^ (rate of change (fall))) above which physiological impacts would lead to irreversible welfare problems. This study provides information on how and to what extent hydropeaking impacts fish communities, guiding the sustainable management of large hydropower plants and the restoration of affected rivers.

**Supplementary Information:**

The online version contains supplementary material available at 10.1007/s10695-026-01633-z.

## Introduction

European and global energy markets have been transformed in recent years, driven by climate change, which has promoted renewable energy sources such as wind, solar, and hydropower (IRENA 2023). Despite being clean in terms of greenhouse gas emissions, these energy sources still impact ecosystems (Sayed et al. 2021). The hydropower industry, for example, often requires large dams and reservoirs that allow for the storage of significant volumes of water, thereby altering the natural flow regime of rivers. Water is channelled towards turbines where energy is generated, resulting in frequent and rapid changes in flow over short periods of time. This phenomenon is known as hydropeaking (Bejarano et al. 2017). Hydropeaking is also associated with alterations to other hydraulic variables, such as water levels, velocity, and turbulence (Greimel et al. 2018). Hydropeaking leads to various ecological impacts on rivers, affecting riparian vegetation (Bejarano et al. 2020; Baladrón et al. 2023), sediment mobilisation (Vericat et al. 2020; López et al. 2023), development of macroinvertebrate communities (Elgueta et al. 2021; Folegot et al. 2021), evolution of algal and periphyton communities (Bondar-Kunze et al. 2016), and fish (Judes et al. 2021). Despite its potential impacts, effective hydrological management can reconcile hydropeaking with the ecological health of rivers, namely *ecopeaking* (Jones 2014). However, *ecopeaking* requires the identification of the thresholds beyond which hydrological alteration leads to irreversible impacts on the ecosystem.

Particular attention has been devoted to the study of fish since early research on the impact of hydropower dams (Bejarano et al. 2018). Numerous reviews focus on species from the *Salmonidae*, followed by the *Cyprinidae*, with less emphasis on other families such as *Centrarchidae*, *Cortidae*, or *Catostomidae* (Bipa et al. 2024). Impacts of hydropeaking on fish are evident at all stages of their life cycle, from the egg phase (Pander et al. 2022) to the adult stage during spawning. In rivers subjected to hydropeaking, multiple ecological impacts on juvenile fish have been reported. Documented effects include stranding events (Auer et al. 2017), alterations in spatial distribution (Rocaspana et al. 2019) and movement patterns (Costa et al. 2019a; Judes et al. 2021), behavioural modifications (Baladrón et al. 2021), changes in body size (Kelly et al. 2016; Rocaspana et al. 2016), and variations in physiological parameters (Bry 1982; Costa et al. 2019b).

Mobilisation of metabolites in fish in stress response induced by hydropeaking has not been thoroughly investigated. Release of these substances is promoted by stressors that significantly activate the release of hormones and other metabolites, which are directly linked to immune system molecules, leading to a loss of immune competence (Yada & Tor 2016). This process is associated with the hypothalamic-pituitary-interrenal (HPI) axis, which induces production and release of steroids such as cortisol from specialised cells in the head kidney, one of the main indicators of physiological stress (Faught et al. 2016; Sopinka et al. 2016). Research on hydropeaking-induced stress has predominantly focused on cortisol and glucose as primary indicators (Flodmark et al. 2002; Earley et al. 2019), often overlooking other metabolites involved in stress responses. These additional biochemical markers include lactate dehydrogenase (LDH), which is released in the event of possible physical injury (Sopinka et al. 2016). Metabolites such as lactate are also considered, as they are indicative of anaerobic metabolism resulting from the muscle activity generated by stress (Dando 1969; Wood et al. 1983). Other markers include nutritional indicators in plasma, such as triglycerides (TGC), which usually reflect the individual’s health status and are released due to increasing energy demands (Wagner & Congleton 2004; Congleton & Wagner 2006). Non-esterified fatty acids (NEFA) are also released with increased energy demands (Henderson & Tocher 1987) and creatine phosphokinase (CPK), whose release is associated with increased anaerobic respiration triggered by muscle activity (Bark & Smith 1982). In addition to these physiological variables, it has been observed that coping with stressful or overstimulating situations can cause changes in fish tissue pigmentation (chromatophores) due to endocrine regulation, potentially serving as an indicator of stress response (Kittilsen et al. 2009; Vissio et al. 2021).

This phenomenon has been documented in several river systems worldwide. For instance, an adverse impact on brook trout (*Salvelinus fontinalis*) communities was observed in the Magpie and Batchawana rivers (Kelly et al. 2017). Similarly, as stated in Korman et al. (2009), it has been documented that the Colorado River’s habitat for rainbow trout (*Oncorhynchus mykiss*) has undergone modifications due to hydropeaking. In Europe, Rocaspana et al. (2019) analysed the effects of hydropeaking-induced water level fluctuations on the mobility patterns of brown trout *(Salmo trutta*). Research on fish conducted in river reaches with hydropeaking has faced several challenges that have complicated the interpretation of results (Alexandre et al. 2023). These involve the dependency on the production scenarios of the power plant and on the specific characteristics of the reaches, difficulties in isolating cause–effect relationships and in the precise monitoring of environmental and biological variables, or dealing with species interactions (Flodmark et al. 2002; Vila-Martínez et al. 2019) and inherent intraspecific variability (Salmaso et al. 2021). As a consequence, many studies have been conducted in hydraulic channels and other experimental infrastructures, which can be tailored to the specific requirements of the study and allow for precise monitoring of variables (Odum 1984; Petersen & Englund 2005). However, indoor hydraulic channels, such as those used in behavioural studies (Baladrón et al. 2021; Costa et al. 2019b), enable the isolation of various environmental factors, but at the cost of an excessive oversimplification of reality.

More complex experimental infrastructures, typically outdoor, recreate a river ecosystem under controlled conditions and are known as mesocosms. These allow for studies that sit between river reaches and indoor channels (Auer et al. 2017; Führer et al. 2024). The use of mesocosms is particularly beneficial for hydropeaking studies due to the possibility of sequentially modifying the hydrological/hydraulic variables associated with the phenomenon, thereby simulating a wide range of hydroelectric production scenarios. Furthermore, mesocosms allow experimentation with healthy fish individuals of similar age and size, thus minimising the influence of initial health status, as well as the effects of interspecific competition and predation, which can amplify the stress response.

Our main aim in this study is to enhance understanding of the effects of hydropeaking on the health of a species of salmonids in order to ensure the welfare of fish communities at the same time as the profitability of hydropower plants. The study will ultimately contribute to the sustainable management of hydropower plants. It was conducted in a river mesocosm, where we simulated different hydroelectric production scenarios (i.e. hydropeaking scenarios) characterised by varying water levels and velocities over time. For each scenario, (1) we quantified the impact of hydropeaking on juvenile rainbow trout (*Oncorhynchus mykiss*), an exotic salmonid species very common in the Iberian Peninsula (Spain), through the levels of specific stress-related physiological variables, and (2) we identified the hydrological/hydraulic environmental variables altered by hydropeaking that influence these stress response variables.

## Materials and Methods

### River mesocosm “Greenchannel”

**Fig. 1:**
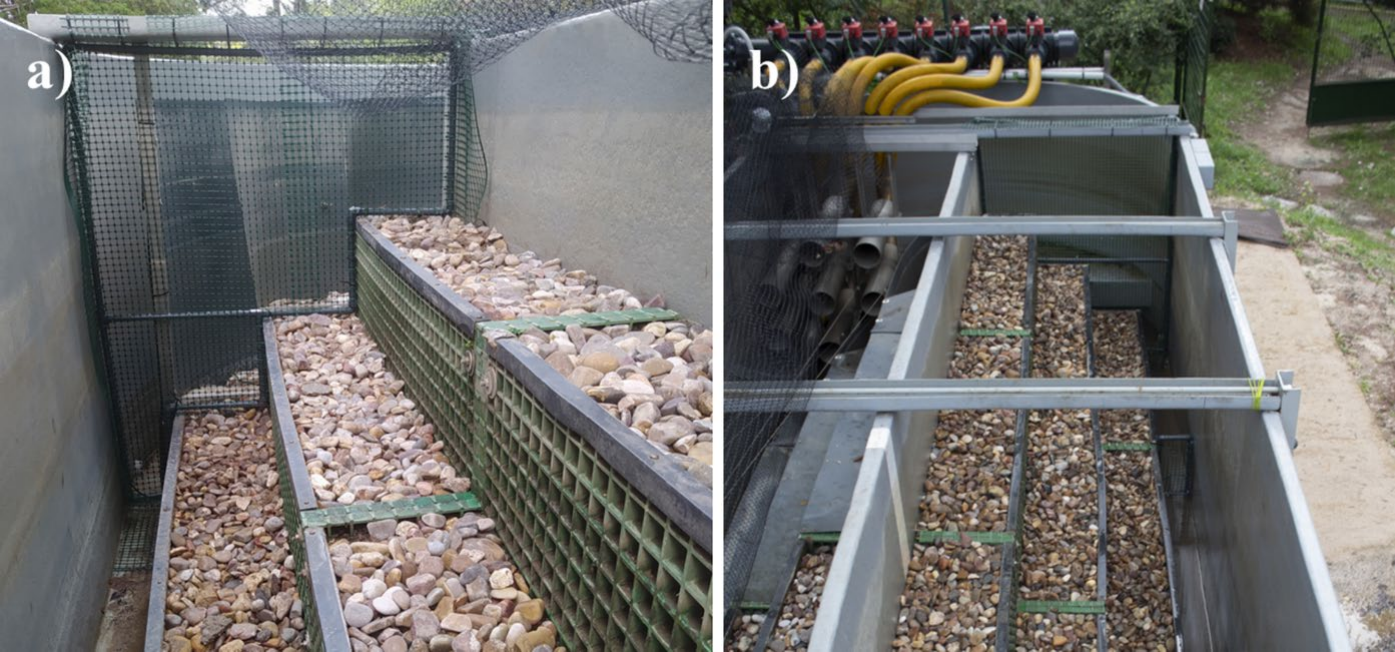
Greenchannel with various adaptations for experimentation with fish. **a** Terraces with a cover of cobbles and gravel and the PVC structure used to confine the trout at one end of the experimental area. **b** The remaining experimental area and the PVC structure used to confine the trout at the other end. Source: Author’s own elaboration

This study was conducted in a river mesocosm called “Greenchannel”, an ecohydrology research facility located at the Forest Engineering School (ETSIMFMN), at the Universidad Politécnica de Madrid (UPM) (Spain). The facility represents a real-scale stream and allows for the simulation of hydromorphological, hydraulic, and ecological phenomena that occur in river ecosystems. The Greenchannel is composed of a set of hydraulic pumps that move water from reservoirs through various nozzles into an oval-shaped channel where it flows. The system allows for the adjustment of water levels and velocities over time according to the specific requirements of the research. Inside the channel, level and velocity sensors provide continuous measurements that are recorded in a Supervisory Control and Data Acquisition system (Miranda®), which manages hydrological scenarios based on the data. Additionally, it includes three terraces at different heights, filled with fluvial, mostly sandy substrate, which replicate different inundation zones. Experimentation was carried out in a delimited area within the Greenchannel measuring 500 cm × 110 cm × 145 cm. Typical salmonid river habitats are characterised by a substrate predominantly composed of coarse particles (cobble and gravel) and a high concentration of dissolved oxygen in the water. To recreate this habitat in the Greenchannel, a surface layer of cobbles and gravel was placed on all terraces, and three aerators were positioned at equidistant points within the experimental area to maintain a constant and adequate oxygen level for trout activity. Also, three thermometers (HOBO Water Temp Pro v2, U22-001 (Bourne, MA, USA)) were placed on each terrace in the middle of the experimental area, which provided continuous information on water temperature. The channel was covered with a semi-translucent mesh to prevent predation and provide shade, which helps reduce the stress response caused by other environmental factors (Karakatsouli et al. 2008) (Fig. 1).

### Velocity mapping

**Fig. 2:**
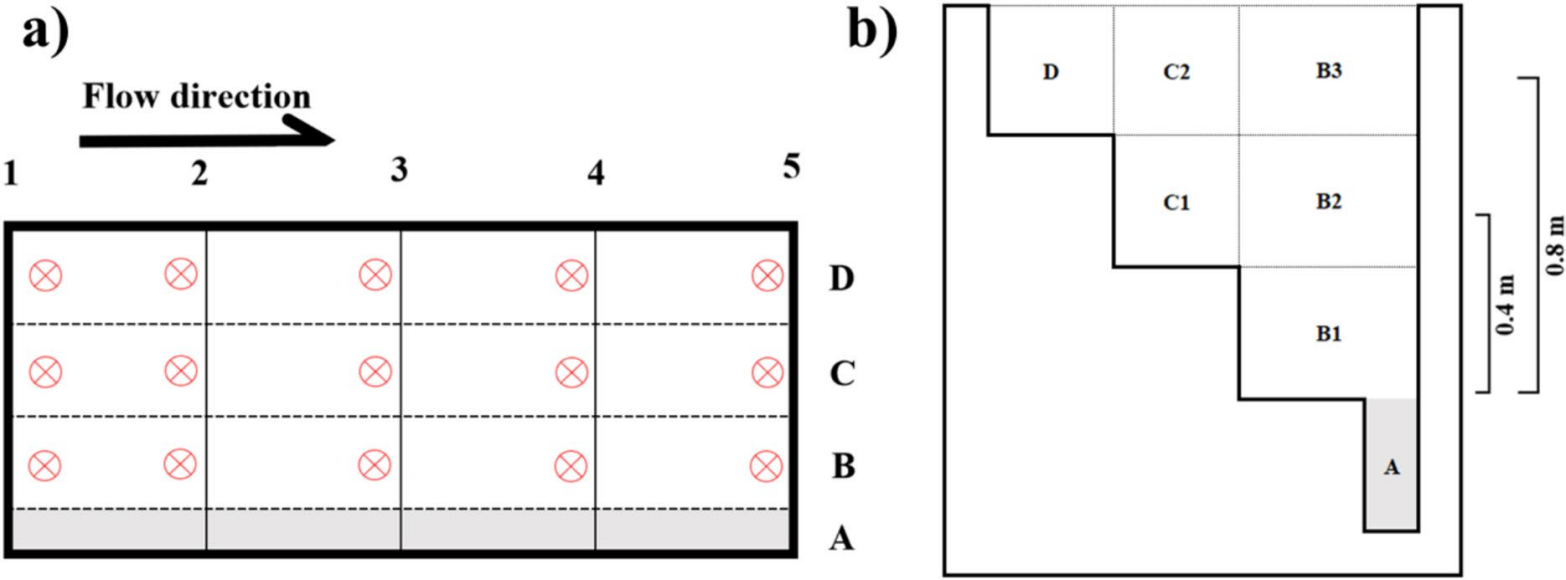
Diagram showing the different points where water velocity measurements were taken within the experimental area delimited in the Greenchannel. **a** Top view indicating the data collection points distributed by numbers (horizontal) and letters (vertical). **b** Side view showing the different depths at which velocity measurements were taken

Current velocity readings were taken at various points within the experimental area under scenarios consisting of combinations of water levels of 1.1 m and 0.7 m and velocities of 0.5 m s^−1^, 0.25 m s^−1^, and 0.1 m s^−1^ using a Valeport Model 801 Electromagnetic Flow Meter (Totnes, UK) (Fig. 2a). After verifying that water velocities were virtually null along the lowest part of the channel (shaded grey in Fig. 2), this area was sealed using a metal mesh that allows water flow but prevents fish access. As a result of this sealing, the effective depth of the channel for experimentation was defined as starting at the level of this mesh and extending to a maximum of 0.8 m (Fig. 2b).

### Fish handling

The selection of the experimental species was predicated on the identification of a salmonid that exhibited analogous ecological and biological characteristics to those of the predominant native species in European rivers like the brown trout. Their ethology is comparable to that of other candidate species, but rainbow trout (*Oncorhynchus mykiss*) are more readily adaptable to experimental conditions.

Ninety juvenile rainbow trout were used, obtained from a commercial fish farm (Fuente Campillo, Cifuentes, Guadalajara), with an average initial weight of 250 g ± 25 g. All individuals were housed at the fish hatchery of the ETSIMFMN at UPM, less than 1 min walking from the Greenchannel. They were first acclimatised for a 2-week period in oxygenated, semi-shaded tanks to reduce any potential stress response in their blood following transport from the original location. For each experimental scenario, 15 different individuals were randomly selected each time and introduced into the Greenchannel for a second acclimatisation period of 5 days, during which a constant water velocity of 0.1 m/s and a depth of 0.4 m were maintained before activating the corresponding experimental scenario. Six scenarios, each lasting 24 h, were run. At the end of each scenario and after analysing the stress variables in the individuals, the fish were returned to the initial tanks at the fish hatchery.

During the first acclimatisation period, fish received commercial feed (EFICO YS 887 F 3 (BioMar) (42% crude protein, 23% crude fat, 4% ash, 2% crude fibre, and 30 ppm astaxanthin)). Feeding was interrupted during the second acclimatisation period in the Greenchannel and during each scenario and was resumed 24 h after the scenario, once the individuals were returned to the fish hatchery (Bermejo-Poza et al. 2016). The degree days of fasting per scenario, based on the sum of the average temperatures for each day, were as follows: control scenario, 87 °C day^−1^; Scenario 2, 58.8 °C day^−1^; Scenario 3, 79.8 °C day^−1^; Scenario 4, 55.8 °C day^−1^; Scenario 5, 47.4 °C day^−1^; and Scenario 6, 60.6 °C day^−1^.

### Experimental design

To design the experimental scenarios simulating different types and intensities of hydropeaking, a literature review was conducted on studies performed in artificial channels and field studies (Table 1). The goal was to quantify the hydrological/hydraulic variables that characterise hydropeaking in experiments with fish, as defined in Bejarano et al. (2017) and Moreira et al. (2019).

**Table 1:** Data compiled from multiple studies involving fish. Flow rate refers to the minimum and maximum water discharge reported in each study, with the value in parentheses indicating the corresponding flow velocity († Natural regime). Fall rate denotes the rate of water level decline. Dashes (-) indicate that the respective parameter was not reported in the study

Species	Type of experiment	Flow rate/velocity	Fall rate (water level or flow)	Reference
*Salmo salar; Salmo trutta*	Regulated natural	*Low* †: 30 m^3^ s^−1^ *High*: 110 m^3^ s^−1^	> 0.8 cm min^−1^	Saltveit et al. (2001)
Juvenile *S. trutta*	Artificial	*Low*: 40 l s^−1^ (0.09–0.13 m s^−1^) *High*: 220 l s^−1^ (0.26–0.33 m s^−1^)	-	Flodmark et al. (2002)
Juvenile *S. trutta*	Regulated natural	*Low* †: 4.9–5.23 m^3^ s^−1^ h^−1^ *High*: 12 m^3^ s^−1^ h^−1^	-	Saltveit et al. (2020)
Juvenile *S. trutta*	Artificial	*Low*: 1 l s^−1^ (< 1 m s^−1^) *High*: 190 l s^−1^ (0.64 m s^−1^)	-	Arnekleiv et al. (2004)
Juvenile *S. trutta*	Artificial	*Low*: 1 l s^−1^ *High*:140 l s^−1^	1–3 cm min^−1^	Halleraker et al. (2003)
*S. trutta*	Artificial	*Low*: 10 l s^−1^ (0.1 m s^−1^) *High*: 220 l s^−1^ (0.8 m s^−1^)	-	Ribi et al. (2014)
*Oncorhynchus mykiss*	Regulated natural	*Low* †: 7 cm s^−1^ *High*: 12 cm s^−1^	-	Korman & Campana (2009)
*Thymallus thymallus*	Artificial	*Low*: 25 l s^−1^ (0.4 m s^−1^) *High*: 400 l s^−1^ (0.8 m s^−1^)	0.5 −3.0 cm min^−1^	Auer et al. (2017)
*S. salar* *S. trutta*	Regulated natural	*Low* †: 6 m^3^ s^−1^ *High*: 20 m^3^ s^−1^	10 m^3^ s^−1^ h^−1^	Vollset et al. (2016)
*S. trutta*	Regulated natural	*Low* †: 0.5 m^3^ s^−1^ *High*: 15 m^3^ s^−1^	-	Rocaspana et al. (2019)

With all the bibliographic information gathered and the boundary conditions of the Greenchannel, six 24-h hydrological scenarios were defined. In each scenario, the following hydrological/hydraulic variables were measured during its 24-h duration: constant water velocity (m s^−1^), maximum water column height (m), daily average rise and fall rate of water levels (m h^−1^), frequency of periods at maximum water level (i.e. inundation; occurrences), and daily average duration of inundation periods (h). Scenario 1 was designated as the control, representing natural river conditions, while Scenarios 2 to 6 simulated different hydropeaking situations, ranging from lower to higher intensity based on the hydrological/hydraulic variables characterising them. These scenarios were programmed into the Miranda® system of the Greenchannel by specifying the required target velocities and water levels for each hour over the 24-h period. The requested velocity-level pair for each hour will be referred to as the “operating instruction” from now on. Table 2 summarises the values of the hydrological/hydraulic variables that characterise each hydrological scenario, and supplementary material (Fig. S.1) shows their graphical representation.

**Table 2:** Hydrological/hydraulic variables and their respective values for each 24-h hydrological scenario (control, natural scenario; scenarios 2–6, hydropeaking scenarios). *V*, Constant water velocity (m s^−1^); *L*_max_, maximum water column level (m); *R*_rise_, daily average water level rise rate (m h^−1^); *R*_fall_, daily average water level fall rate (m h^−1^); *F*_inund._, frequency of periods at maximum water level (i.e. inundation; occurrences per day); *D*_inund._, daily average of inundation periods (h)

	*V* (m s^−1^)	*L*_max_ (m)	*R*_rise_ (m h^−1^)	*R*_fall_ (m h^−1^)	*F*_inund_ (#)	*D*_inund_ (h)
Control	0.1	0.4	0	0	0	0
Scenario 2	0.25	0.4	0	0	0	0
Scenario 3	0.25	0.8	0.25	0.133	1	5
Scenario 4	0.5	0.8	0.25	0.133	1	5
Scenario 5	0.5	0.8	0.25	1.2	1	5
Scenario 6	0.5	0.8	0.25	1.2	3	1

### Water quality analysis

For the purpose of this study, we used water sourced from the ETSIMFMN well. Prior to the experiments, water quality analysis was conducted in collaboration with the Department of Civil Engineering: Hydraulics, Energy, and Environment at UPM, following the physicochemical standards outlined in the EU Water Framework Directive (Directive 2000/60/EC). Laboratory-measured variables included temperature, pH, conductivity, dissolved oxygen, turbidity, nitrates (NO_3_^−^), nitrites (NO_2_^−^), phosphates (PO_4_^3−^), suspended solids, and dissolved solids. Temperature, pH, conductivity, and dissolved oxygen were measured using portable probes. Turbidity was assessed with a Hanna HI93703 infrared turbidimeter (Leighton Buzzard, UK), employing the nephelometric method. Measurements were reported in nephelometric turbidity units (NTU), equivalent to formazin turbidity units (FTU) as defined by the legislation. Nitrates, nitrites, and phosphates were analysed using cuvette tests with a Spectroquant NOVA 60 photometer (Darmstadt, Germany). Suspended and dissolved solids were determined using the gravimetric method (American Public Health Association 2023).

Additionally, water quality was analysed once for each scenario, 3 h after its initiation. Measurements included water temperature, dissolved oxygen using a portable ProfiLine Oxi 3310 oxygen metre (Burlington, MA, USA), and turbidity using the same turbidimeter employed in the initial analysis.

### Sample collection

After each 24-h scenario, the corresponding 15 trout were crowded using nets and harvested with landing nets (Arnekleiv et al. 2004). To minimise stress response during handling, individuals were anesthetised in a tub containing eugenol (0.1 ml per litre of water). This practice is known to reduce fish activity, keeping them sedated and thereby decreasing the release of stress-related hormones and compounds (Guénette et al. 2007).

Once sedated, blood samples (~ 2 ml per fish and scenario) were collected intravenously via puncture of the caudal vein. Samples were centrifuged at 6000 rpm for approximately 5 min to separate plasma, which was stored and maintained at 4 °C until analysis. Simultaneously, dorsal pigmentation was measured following the methodology of Colihueque et al. (2011) using a Minolta Spectrophotometer CM-2500c (Tokyo, Japan). Measurements were based on the CIELab* colour space (Commission Internationale de l’Eclairage, CIE) (CIE 1977), recording red (a*), yellow (b*), and lightness (L*) index. From these, chroma (C* = (a^2^ + b^2^)^1/2^) and hue (h* = arctan(b*/a*) × 57.29) values were calculated. Each fish was weighed and measured from the mandibular symphysis to the base of the dorsal lobe of the caudal fin. The procedure for each individual took no more than 30 s to avoid distress and potential electrolyte imbalances that could harm the fish (Rozas et al. 2016). In the laboratory, physiological variables were analysed, including substrates such as cortisol, lactate, triglycerides (TGC), and non-esterified fatty acids (NEFA), as well as enzymes like creatine phosphokinase (CPK) and lactate dehydrogenase (LDH). Other substrates, such as adrenaline and glucose, were not selected for measurement. From now on, these substrates and the histological pigmentation variables are collectively referred to as physiological variables.

### Assay procedures

We examined the plasma concentrations of cortisol, lactate, TGC, NEFA, and the enzymes LDH and CPK. Cortisol concentrations were determined through an enzyme immunoassay using a Cortisol EIA kit provided by Radim Ibérica S.A. (Barcelona, Spain). Lactate levels were measured employing enzymatic-spectrophotometric techniques supplied by Spinreact S.A. (Sant Erece de Bas, Spain). TGC were assessed using an enzymatic method with a Boehringer Mannheim Kit (Barcelona, Spain). NEFA levels were quantified through an enzymatic-colorimetric method using Randox Diagnostic kits (London, UK). LDH activity was determined according to the method described by Furné et al. (2012), which involves the oxidation of NADH during pyruvate’s conversion to lactate. CPK concentrations were measured using a Roche/Hitachi Chemistry Analyzer (Roche Diagnostics, S.L., Sant Cugat del Vallès, Spain), based on the conversion of creatinine phosphate into creatinine at 340 mm.

### Data analysis

Physiological variables and water quality metrics were compared between the control scenario and hydropeaking scenarios 2 through 6, as well as across different hydrological/hydraulic variables. For physiological variables, statistical significance was evaluated using the non-parametric Kruskal–Wallis test followed by pairwise Wilcoxon post-hoc tests with Bonferroni correction. For physicochemical water quality variables, linear or exponential functions were fitted to the data, and their *R*^2^ values were computed.

Principal component analysis (PCA) was performed on the physiological variables to identify those most sensitive to hydropeaking and potential correlations among them. Generalised linear models (GLM) were developed to explain the linear relationships between physiological response variables and the hydrological/hydraulic scenario characterisation variables. In all models, Gaussian probability distribution and identity link function were assumed. All hydrological/hydraulic variables were included as predictors in the GLM, and a stepwise backward variable selection procedure was applied to obtain a final set of predictors that minimised the Akaike information criterion (AIC). Variables were introduced into the models without standardisation to generate equations useful for prediction (Vittinghoff et al. 2012).

Given that the rise rate of water level has not been identified in previous studies as critical for fish species (Greimel et al. 2018), this study focuses solely on the effect of the fall rate of water level (responsible for stranding mortality; Auer et al. 2017), referred to hereafter as the rate of change. In our experimental design, frequency and duration of the highest water level (0.8 m; referred to as inundation in this text) are inversely related (increased inundation frequency over 24 h corresponds to decreased duration). Therefore, the effect of these two variables on fish was assessed through a single variable derived from their ratio (frequency/duration; # h^−1^). Therefore, hydrological/hydraulic variables used in the statistical analyses were velocity (m s^−1^), level (m), rate of change (fall; m h^−1^), and the ratio of inundation frequency to duration (frequency/duration; # h^−1^). Statistical analyses were conducted using R software version 4.3.2 (R Core Team 2024).

### Ethical statement

All procedures involving animal handling and care were conducted in accordance with European standards and Spanish legislation (Directive 2010/63/EU; Ley 6/2013; Real Decreto 53/2013). A permit was issued to the responsible entities, the Universidad Politécnica de Madrid and Universidad Complutense de Madrid, to ensure compliance with these regulations. No fish were sacrificed for the purposes of this study. The principles of replacement, reduction, and refinement (3Rs) were adhered to throughout the experimental design.

## Results

Results are divided into three sections. The “Water velocity” section presents the outcomes of water velocity mapping in the Greenchannel to evaluate variability within the experimental area under the same scenario. The “Water quality” section shows the findings from water quality analyses. The “Physiological variables in trout” section details the physiological responses of trout under different hydrological scenarios and their relation to the hydrological/hydraulic variables characterising those scenarios.

### Water velocity

**Fig. 3:**
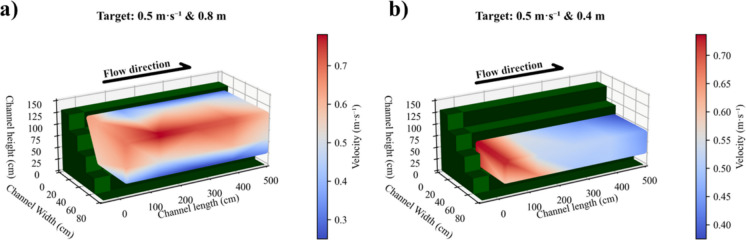
Spatial representation of velocity data (measured and extrapolated) for an example of two different operating instructions (pairs velocity-level). **a** Operating instruction: 0.5 m s^−1^ velocity and 0.8 m water level. **b** Operating instruction: 0.5 m s^−1^ velocity and 0.4 m water level

Mean velocities and standard deviations for all measurements taken under each operating instruction (pairs velocity-level) are provided (Table 3). For a water level of 0.4 m and velocities of 0.1, 0.25, and 0.5 m s^−1^, average velocities within the experimental area were 0.073 ± 0.015, 0.236 ± 0.028, and 0.485 ± 0.089 m s^−1^, respectively. For a water level of 0.8 m and velocities of 0.1, 0.25, and 0.5 m s^−1^, average velocities within the experimental area were 0.141 ± 0.020, 0.224 ± 0.035, and 0.496 ± 0.059 m s^−1^, respectively. Using the Cartesian coordinates of measurement points, a 3D plot (Fig. 3; Fig. S.2) represents the measured velocity values and extrapolated velocity distributions across the experimental section for each operating instruction.

**Table 3:** Measured velocities (and standard deviation) for each operating instruction (i.e. requested target velocity-level pairs

Target water level (m)	Target velocity (m s^−1^)	Measured velocity (m s^−1^)
0.4	0.10	0.073 ± 0.015
	0.25	0.236 ± 0.028
	0.50	0.485 ± 0.089
0.8	0.10	0.141 ± 0.020
	0.25	0.224 ± 0.035
	0.50	0.496 ± 0.059

For all tested operating instructions, the observed velocities in the beginning of the experimental area and on the right-hand side of the experimental area were slightly higher than the programmed velocity. In contrast, at the bottom of the channel and in areas adjacent to the terraces, velocities were slightly lower than the programmed velocity (Fig. 3; Fig. S.2).

### Water quality

Pre-experiment, deep water quality analysis showed a pH of 7.19, a conductivity of 643 μS cm^−1^, 9.29 mg of O_2_ l^−1^, and 3.02 NTU of turbidity. Additionally, the water presented a concentration of 5 mg l^−1^ of nitrates, 0.041 mg l^−1^ of nitrites, and a concentration of < 0.5 mg l^−1^ of phosphates. The gravimetric method showed a concentration of 6 mg l^−1^ of total suspended solids and 454 mg l^−1^ of total dissolved solids.

Water quality analyses conducted for each scenario are summarised in Table 4. Temperatures ranged from 14.5 °C in the control scenario to 7.9 °C in scenario 5 (Fig. S.3). A strong relationship was observed between temperature and water velocity (*R*^2^ = 0.72), and a somewhat weaker correlation with hydropeaking intensity (*R*^2^ = 0.48) (Fig. S.3a and b). Dissolved oxygen levels ranged from 8 mg (O₂) l^−1^ (scenario 3) to 12 mg (O₂) l^−1^ (scenario 2), with no significant relationship to hydropeaking intensity or any associated hydrological/hydraulic variables (Fig. S.4). Finally, turbidity increased with the intensity of hydropeaking (Fig. S.5a), as higher intensities involved scenarios with greater velocities (*R*^2^ = 0.58; Fig. S.5b), higher fall rates (*R*^2^ = 0.67; Fig. S.5c), and greater variations in water levels (*R*^2^ = 0.83; Fig. S.5d). The lowest turbidity was observed in scenario 2, with 2.20 NTU, while the highest was found in scenario 6, with 15.75 NTU.

**Table 4:** Water temperature, dissolved oxygen, turbidity, and maximum ambient temperature (on the day of water sampling) for each hydrological scenario

	Maximum air temperature (°C)	Water temperature (°C)	Dissolved oxygen (mg(O_2_) l^−1^)	Turbidity (NTU)
Control	14.00	14.50	10.10	3.02
Scenario 2	12.00	9.80	12.80	2.20
Scenario 3	10.00	12.30	8.06	4.43
Scenario 4	16.00	9.30	11.45	6.78
Scenario 5	10.00	7.90	9.60	7.89
Scenario 6	7.00	10.10	8.85	15.72

### Physiological variables in trout

The average weight of individuals following exposure to the scenarios was 278.90 ± 9.20 g, with a mean length of 28.99 ± 0.33 cm.

#### Differences between hydropeaking scenarios and hydrological/hydraulic variables

Table 5 presents the mean values and standard deviations for lightness, cortisol, lactate, TGC, LDH, CPK, and NEFA obtained in each hydrological scenario, along with their respective significance levels. Among the histological pigmentation variables, only lightness showed statistically significant differences between scenarios, leading to the exclusion of C*, h*, a*, and b* from further statistical analyses and figures (Tables S.1 and S.2). The remaining physiological variables exhibited significant differences between scenarios (*p*-values below 0.05).

**Table 5:** Physiological variables obtained across all hydrological scenarios (mean and standard deviation) and the significance level determined using the non-parametric Kruskal–Wallis test between scenarios. “n” indicates the number of trout used in the experiment. *Sc*, scenario; *TGC*, triglycerides; *LDH*, lactate dehydrogenase; *CPK*, creatine phosphokinase; *NEFA*, non-esterified fatty acids

Variables	Control (*n* = 14)	Sc. 2 (*n* = 15)	Sc. 3 (*n* = 14)	Sc. 4 (*n* = 14)	Sc. 5 (*n* = 15)	Sc. 6 (*n* = 15)	*p*-value
Lightness	54.51 ± 6.78	63.98 ± 5.22	59.10 ± 5.43	61.59 ± 6.94	64.81 ± 6.39	64.71 ± 9.33	< 0.001
Cortisol (ng ml^−1^)	14.12 ± 5.08	9.40 ± 4.02	5.31 ± 4.11	10.62 ± 2.96	5.41 ± 4.18	2.26 ± 3.05	< 0.001
Lactate (mmol l^−1^)	36.10 ± 7.02	20.73 ± 6.84	26.93 ± 5.93	16.52 ± 3.94	24.99 ± 3.75	22.70 ± 4.76	< 0.001
TGC (mg dl^−1^)	126.93 ± 21.62	138.66 ± 19.44	137.86 ± 22.75	125.21 ± 16.23	172.33 ± 38.47	148.53 ± 31.67	< 0.001
LDH (U l^−1^)	513.36 ± 435.96	658.73 ± 484.55	427.14 ± 425.45	312.93 ± 196.42	320.8 ± 185.96	429.4 ± 174.13	0.004
CPK (U l^−1^)	38.00 ± 20.90	132.40 ± 133.50	80.93 ± 48.05	108.21 ± 259.45	170.27 ± 166.98	131.00 ± 144.32	< 0.001
NEFA (mmol l^−1^)	0.29 ± 0.04	0.37 ± 0.10	0.29 ± 0.07	0.38 ± 0.10	0.35 ± 0.08	0.33 ± 0.07	0.5206

**Fig. 4:**
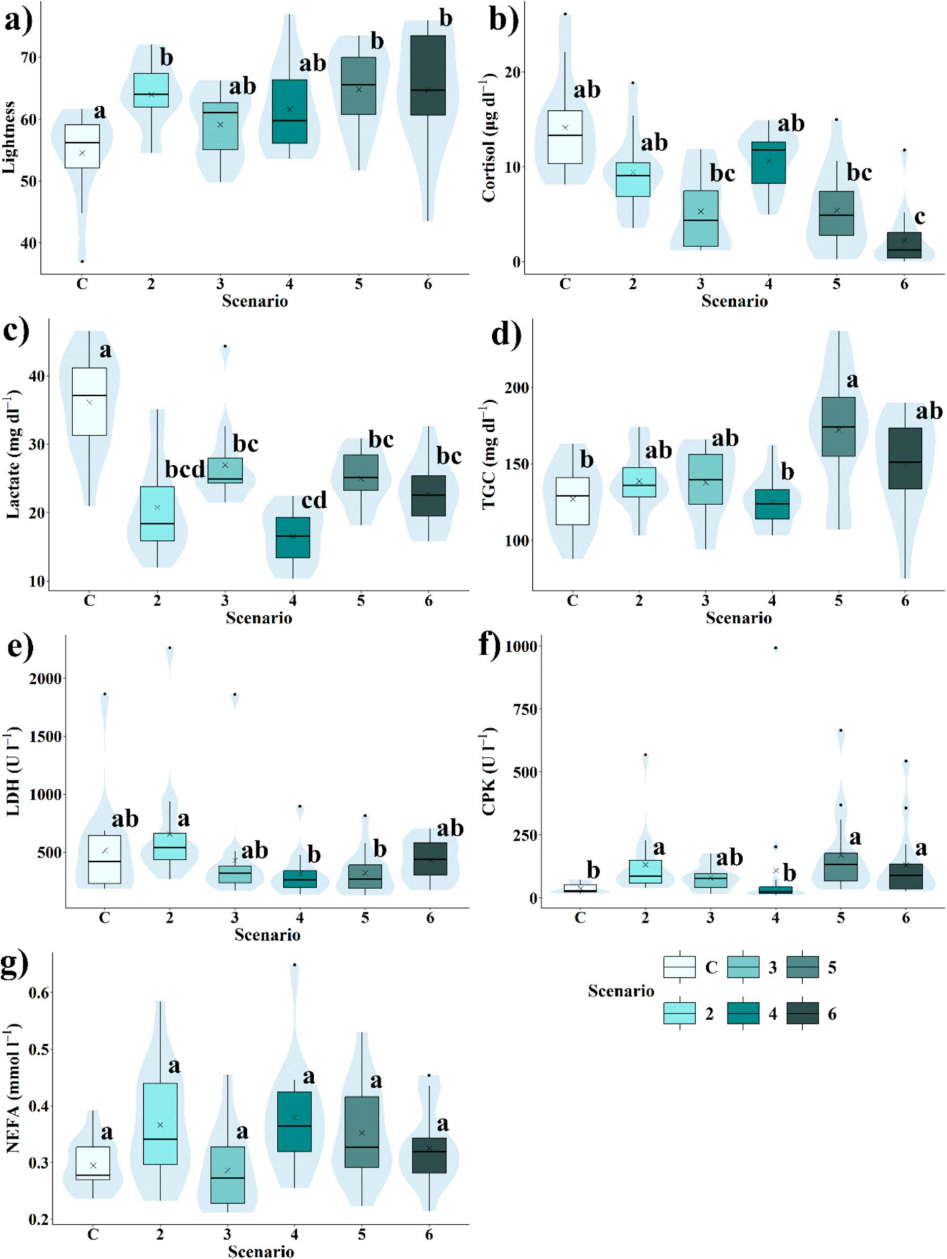
Box-and-whisker plots representing physiological variables (lightness, cortisol, lactate, triglycerides, LDH, CPK, and NEFA) measured across the different hydrological scenarios (*C*, control scenario). Significant differences between experimental scenarios are indicated by letters a, b, and c according to the results of pairwise post-hoc analysis (*p* < 0.05). The arithmetic mean for each scenario is indicated by x. The central lines indicate, from bottom to top, the first quartile (25%), the median (50%), and the third quartile (75%) of the data. Whiskers extend to the furthest data points within the range, excluding outliers which are represented by dots

**Fig. 5:**
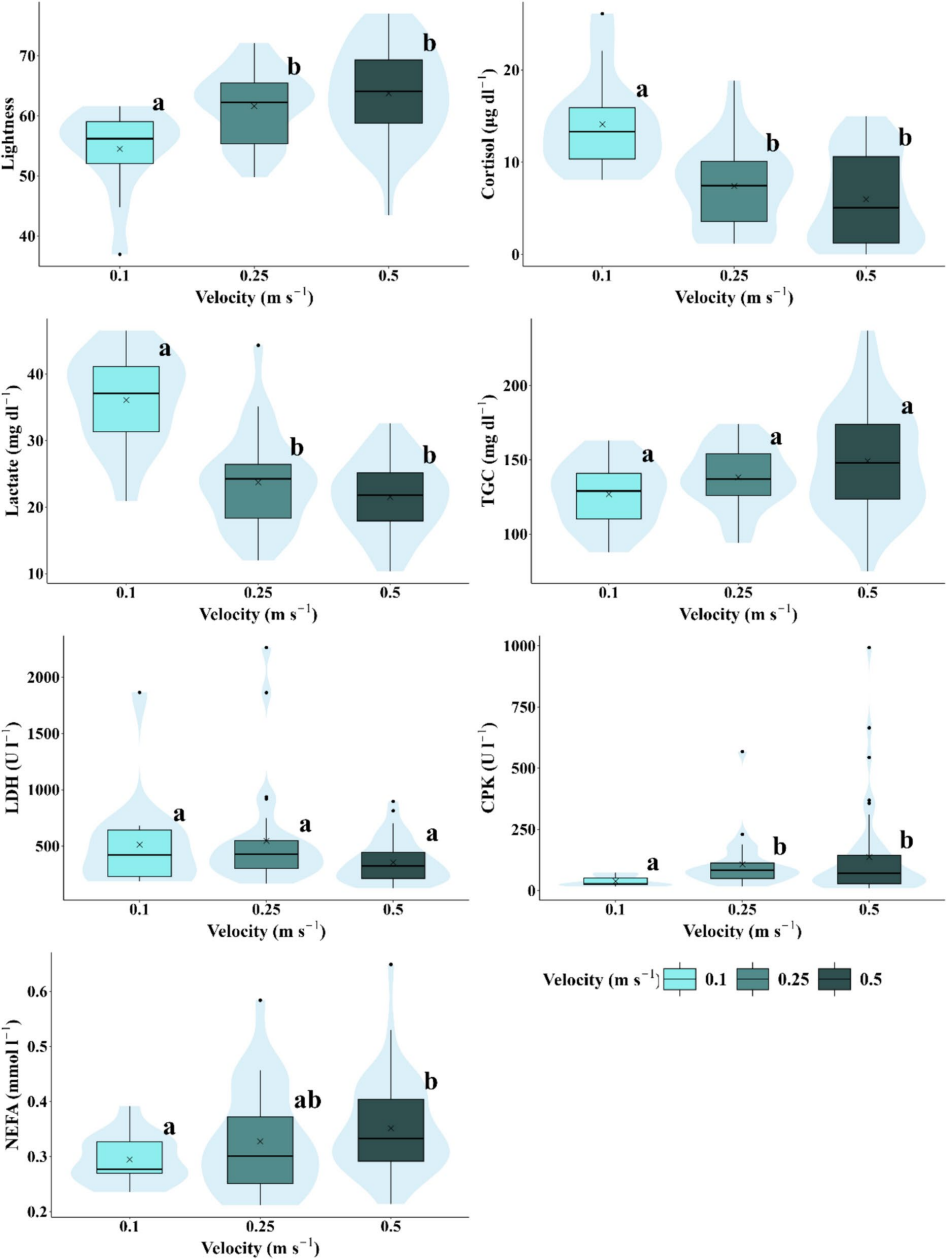
Box-and-whisker plots representing the response variables measured (lightness, cortisol, lactate, triglycerides, LDH, CPK, and NEFA) according to flow velocity. Significant differences between experimental scenarios are indicated by different letters (a, b, and c) based on the results of the pairwise post-hoc analysis (*p* < 0.05). The arithmetic mean for each scenario is marked with an x. The central lines represent, from bottom to top, the first quartile (25%), the median (50%), and the third quartile (75%) of the data. Whiskers on either side of the box extend to the furthest data points within the non-outlier range, while outliers are depicted as individual points

**Fig. 6:**
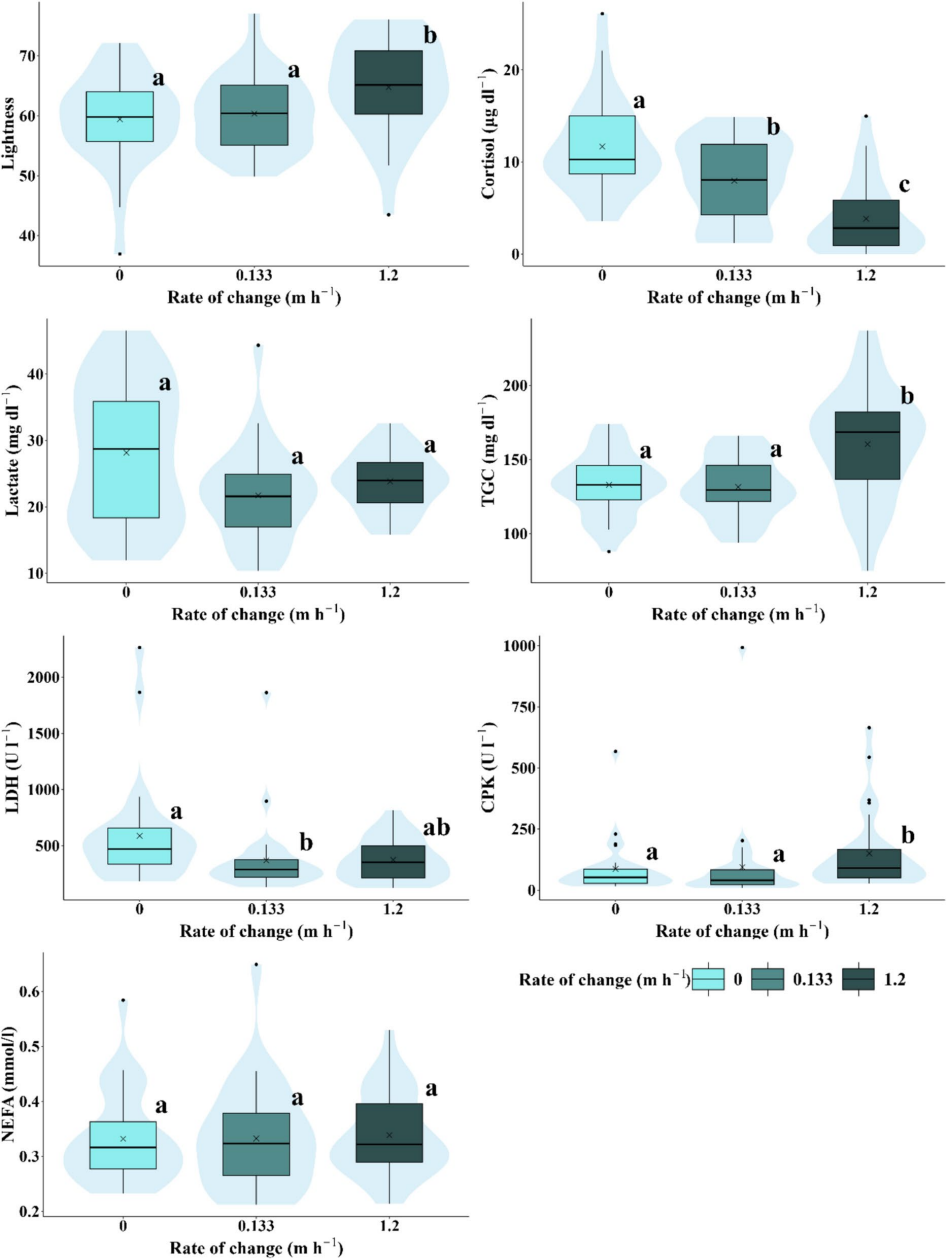
Box-and-whisker plots representing the response variables measured (lightness, cortisol, lactate, triglycerides, LDH, CPK, and NEFA) according to rates of change. Significant differences between experimental scenarios are indicated by different letters (a, b, and c) based on the results of the pairwise post-hoc analysis (*p* < 0.05). The arithmetic mean for each scenario is marked with an x. The central lines represent, from bottom to top, the first quartile (25%), the median (50%), and the third quartile (75%) of the data. Whiskers on either side of the box extend to the furthest data points within the non-outlier range, while outliers are depicted as individual points

**Fig. 7:**
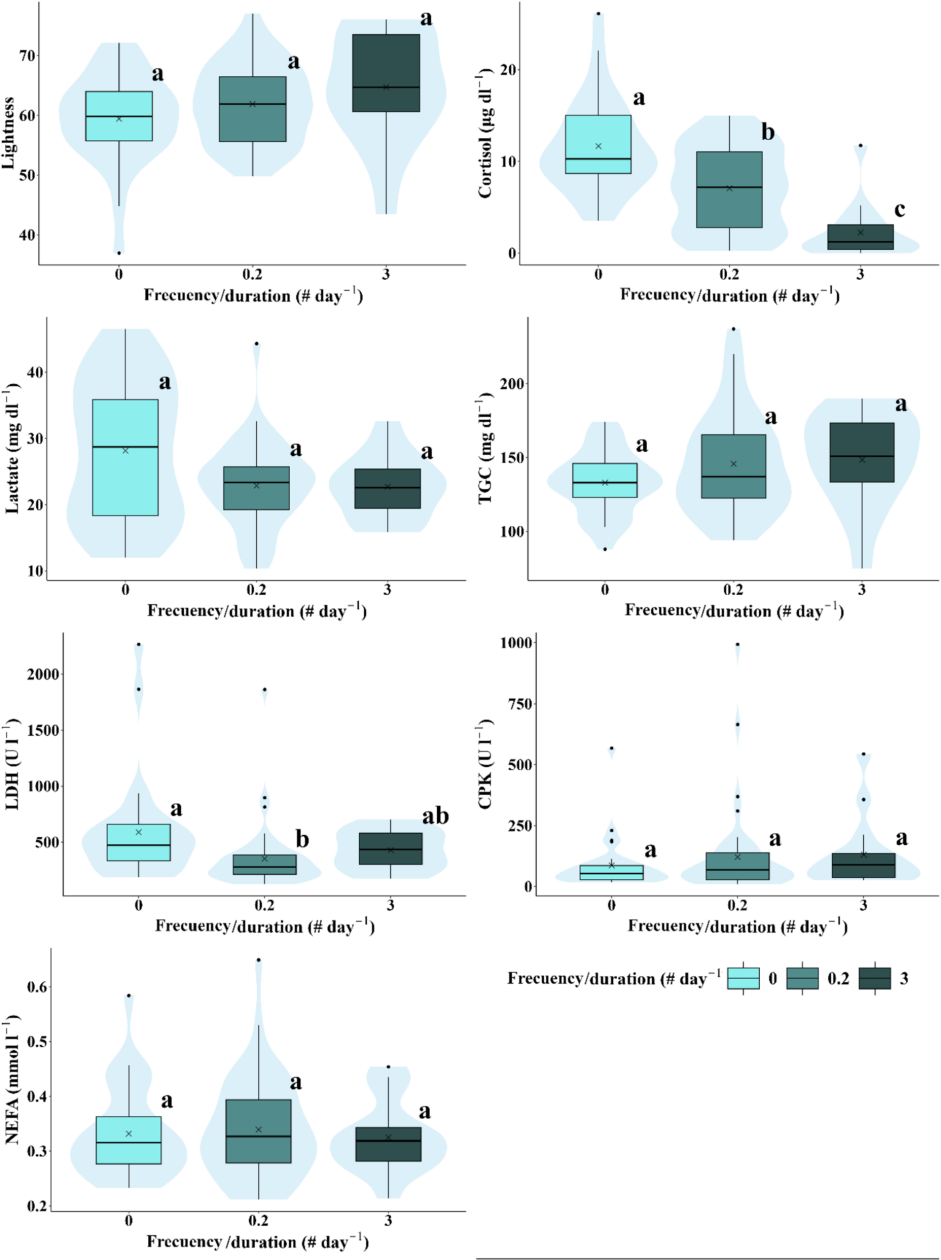
Box-and-whisker plots representing the response variables measured (lightness, cortisol, lactate, triglycerides, LDH, CPK, and NEFA) according to inundation frequency/duration ratios. Significant differences between experimental scenarios are indicated by different letters (a, b, and c) based on the results of the pairwise post-hoc analysis (*p* < 0.05). The arithmetic mean for each scenario is marked with an x. The central lines represent, from bottom to top, the first quartile (25%), the median (50%), and the third quartile (75%) of the data. Whiskers on either side of the box extend to the furthest data points within the non-outlier range, while outliers are depicted as individual points

**Fig. 8:**
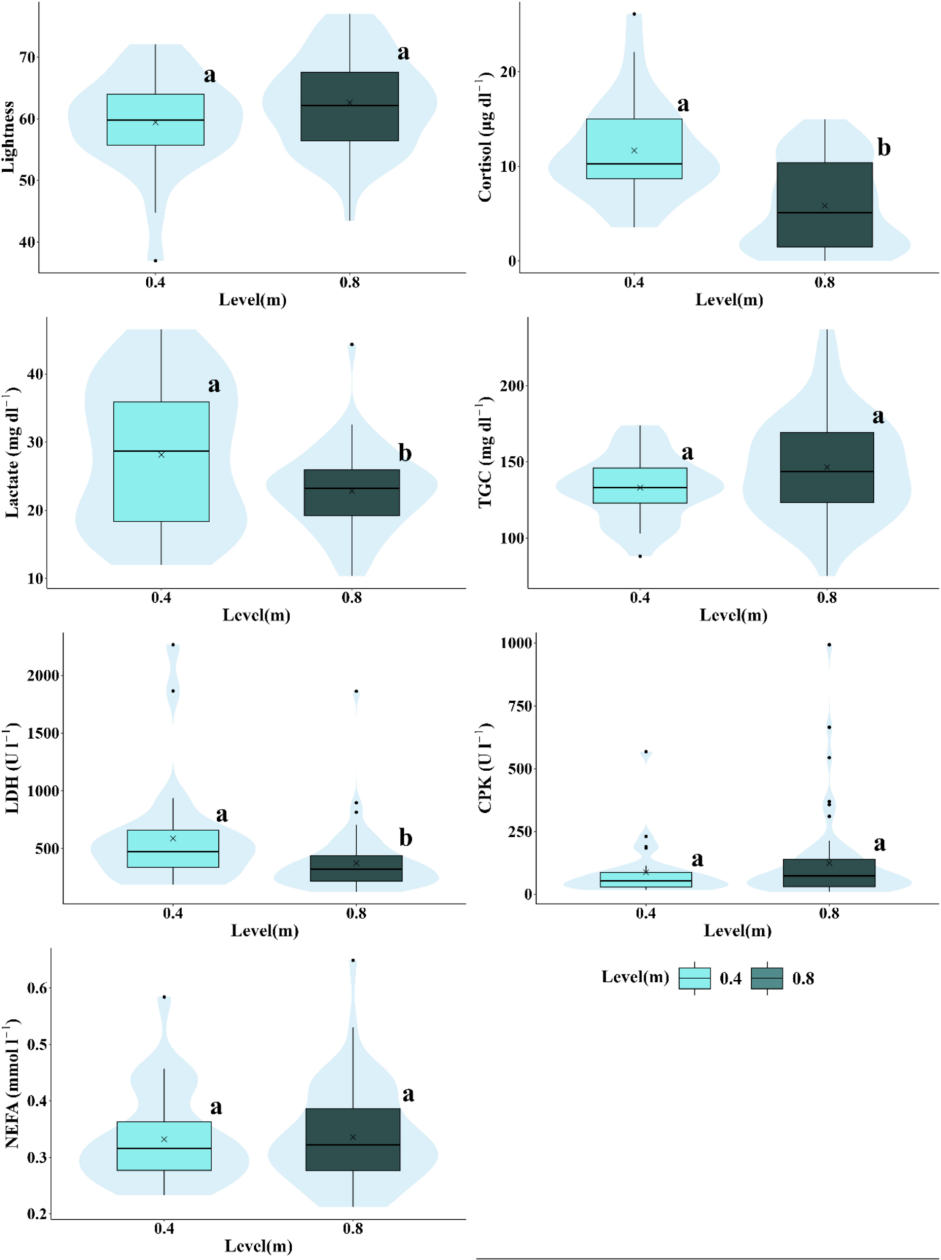
Box-and-whisker plots representing the response variables measured (lightness, cortisol, lactate, triglycerides, LDH, CPK, and NEFA) according to the maximum inundation level. Significant differences between experimental scenarios are indicated by different letters (a, b, and c) based on the results of the pairwise post-hoc analysis (*p* < 0.05). The arithmetic mean for each scenario is marked with an x. The central lines represent, from bottom to top, the first quartile (25%), the median (50%), and the third quartile (75%) of the data. Whiskers on either side of the box extend to the furthest data points within the non-outlier range, while outliers are depicted as individual points

Overall, lightness increased with hydropeaking intensity (Fig. 4a), but there were no statistically significant differences between Scenarios 2, 3, 4, 5, and 6, while the control scenario differed significantly from Scenarios 2, 5, and 6. For the different hydrological/hydraulic variables, lightness increased with velocity, with statistically significant differences observed between scenarios with a velocity of 0.1 m s^−1^ compared to other velocity scenarios (Fig. 5). Regarding the rate of change, significant differences in this variable were only observed in scenarios with a rate of change of 1.2 m h^−1^ (Fig. 6), whereas there were no significant differences between water levels or the frequency/duration ratio of the inundation (Table 6; Figs. 7 and 8).

**Table 6:** Average and standard deviations for physiological variables (Lightness shown exclusively for pigmentation) in relation to hydrological/hydraulic variables potentially impacted by hydropeaking which characterised the hydrological scenarios. Significant differences between groups are indicated by letters (a, b, c) (*p* < 0.05)

	Velocity (m s^−1^)			Rate of change (m h^−1^)			Frequency/duration (# h^−1^)			Level (m)	
	0.1	0.25	0.50	0	0.133	1.2	0	0.2	3	0.4	0.8
Lightness	54.5 ± 6.78^a^	61.6 ± 5.79^b^	63.8 ± 7.6^b^	59.4 ± 7.63^a^	60.6 ± 6.25^a^	64.8 ± 7.85^b^	59.4 ± 7.63^a^	61.9 ± 6.58^a^	64.7 ± 9.33^a^	59.4 ± 7.63^a^	62.6 ± 7.40^a^
Cortisol (ng ml^−1^)	14.1 ± 5.08^a^	7.42 ± 4.50^b^	5.99 ± 4.8^b^	11.7 ± 5.09^a^	7.9 ± 4.44^b^	3.8 ± 3.9^c^	11.7 ± 5.08^a^	7.1 ± 4.47^b^	2.26 ± 3.05^c^	11.7 ± 5.08^a^	5.83 ± 4.64^b^
Lactate (mmol l^−1^)	36.1 ± 7.02^a^	23.7 ± 7.04^b^	21.5 ± 5.4^b^	28.2 ± 10.4^a^	21.7 ± 7.25^a^	23.9 ± 4.37^a^	28.6 ± 10.4^a^	22.9 ± 6.40^a^	22.7 ± 4.76^a^	28.2 ± 10.36^a^	22.8 ± 5.98^b^
TGC (mg dl^−1^)	126.9 ± 21.6^a^	138.3 ± 20.72^a^	149.3 ± 35.5^a^	133.0 ± 21.0^a^	131.5 ± 20.4^a^	160.4 ± 36.7^b^	133.0 ± 21.0^a^	145.8 ± 33.9^a^	148.5 ± 31.7^a^	133.0 ± 21.02^a^	146.5 ± 33.08^a^
LDH (U l^−1^)	513.4 ± 436.0^a^	546.9 ± 464.0^a^	355.3 ± 188.9^a^	588.6 ± 459.5^a^	370.0 ± 330.3^b^	375.1 ± 185.4^ab^	588.6 ± 459.5^a^	352.9 ± 286.77^b^	429.4 ± 174.1^ab^	588.6 ± 459.5^a^	372.7 ± 263.0^b^
CPK (U l^−1^)	38.0 ± 20.9^a^	107.6 ± 103.3^b^	137.1 ± 192.0^b^	86.8 ± 106.9^a^	94.6 ± 183.6^a^	150.6 ± 154.7^b^	86.8 ± 106.9^a^	120.9 ± 179.7^a^	131.0 ± 144.3^a^	86.8 ± 106.9^a^	123.6 ± 170.1^a^
NEFA (mmol l^−1^)	0.29 ± 0.04^a^	0.33 ± 0.09^ab^	0.35 ± 0.08^b^	0.33 ± 0.08^a^	0.33 ± 0.10^a^	0.34 ± 0.07^a^	0.33 ± 0.08^a^	0.34 ± 0.09^a^	0.32 ± 0.07^a^	0.33 ± 0.08^a^	0.33 ± 0.08^a^

Cortisol levels were significantly higher in the control and Scenario 4 compared to Scenarios 3, 5, and 6, with these three being similar to each other (Fig. 4b), whereas Scenario 2 did not show significant differences from any other scenario. There was a decreasing trend as hydropeaking intensity decreased, with the highest levels recorded in the control and the lowest in Scenario 6. Cortisol levels in individuals exposed to high velocities, rate of change, water levels, and inundation frequency/duration ratios were significantly lower (Table 6; Figs. 5, 6, and 8). Notably, at a velocity of 0.1 m s^−1^, levels of this parameter were significantly higher than at higher velocities of 0.25 and 0.5 m s^−1^, with no significant differences observed between the latter two (Table 6).

The general trend observed in plasma lactate levels showed a decrease with increasing hydropeaking intensity. Lactate levels in the control scenario differed significantly from those in all other experimental scenarios. Levels in Scenarios 3, 5, and 6 were similar to each other, as were those in Scenarios 2 and 4, though Scenario 2 did not show significant differences compared to other scenarios (Fig. 4c). Similar to cortisol and lightness, lactate levels decreased with increasing water velocity, but this was only significant between 0.1 and 0.25 m s^−1^. No significant differences were observed in concentration concerning other hydrological/hydraulic variables, except for water level, where concentration decreased when the water level rose to 0.8 m (Table 6).

TGC levels were similar across the control, 2, 3, 4, and 6 scenarios but increased significantly in Scenario 5, showing higher levels compared to the control and Scenario 4 (Fig. 4d). No differences in this variable were found between velocity and frequency/duration ratio scenarios. However, significant differences were observed for rate of change scenarios, with TGC levels increasing in individuals exposed to the highest rate (1.2 m h^−1^) compared to low (0.133 m h^−1^) and null rates (Table 6).

LDH levels were significantly higher in Scenario 2 compared to Scenarios 4 and 5. No significant differences were observed among the other scenarios (Fig. 4e). LDH levels across velocity scenarios were similar. An increase in water level from 0.4 to 0.8 m significantly reduced concentration. In scenarios involving the rate of change and frequency/duration of inundation, LDH levels were higher under null rate and null frequency/duration compared to the other rates and frequencies/durations. However, significant differences were only observed between the null and medium rates (0.133 m h^−1^) and between the null and medium frequency/duration ratios.

CPK levels in individuals from Scenario 3 were similar to those in the other hydropeaking scenarios. CPK levels in the control and Scenario 4 were similar, as were those in Scenarios 2, 5, and 6, but significant differences were observed between these two groups. CPK levels in the control and Scenario 4 were significantly lower than in Scenarios 2, 5, and 6 (Fig. 4f). Concentration increased with velocity, but significant differences were only observed between velocities of 0.1 and 0.25 m s^−1^. Increasing the rate of change also led to higher CPK levels, with significant differences found between rates of 0.133 and 1.2 m h^−1^. CPK levels increased with frequency/duration ratios and water levels, but these changes were not statistically significant (Table 6).

NEFA levels showed no significant differences across hydropeaking scenarios (Fig. 4g) or most hydrological/hydraulic variables. The only exception was a significant increase in NEFA levels with velocity, from 0.1 to 0.5 m s^−1^, although no significant differences were found between 0.1 and 0.25 m s^−1^ or between 0.25 and 0.5 m s^−1^ (Fig. 4g; Table 6).

#### Correlation and principal component analysis (PCA)

**Fig. 9:**
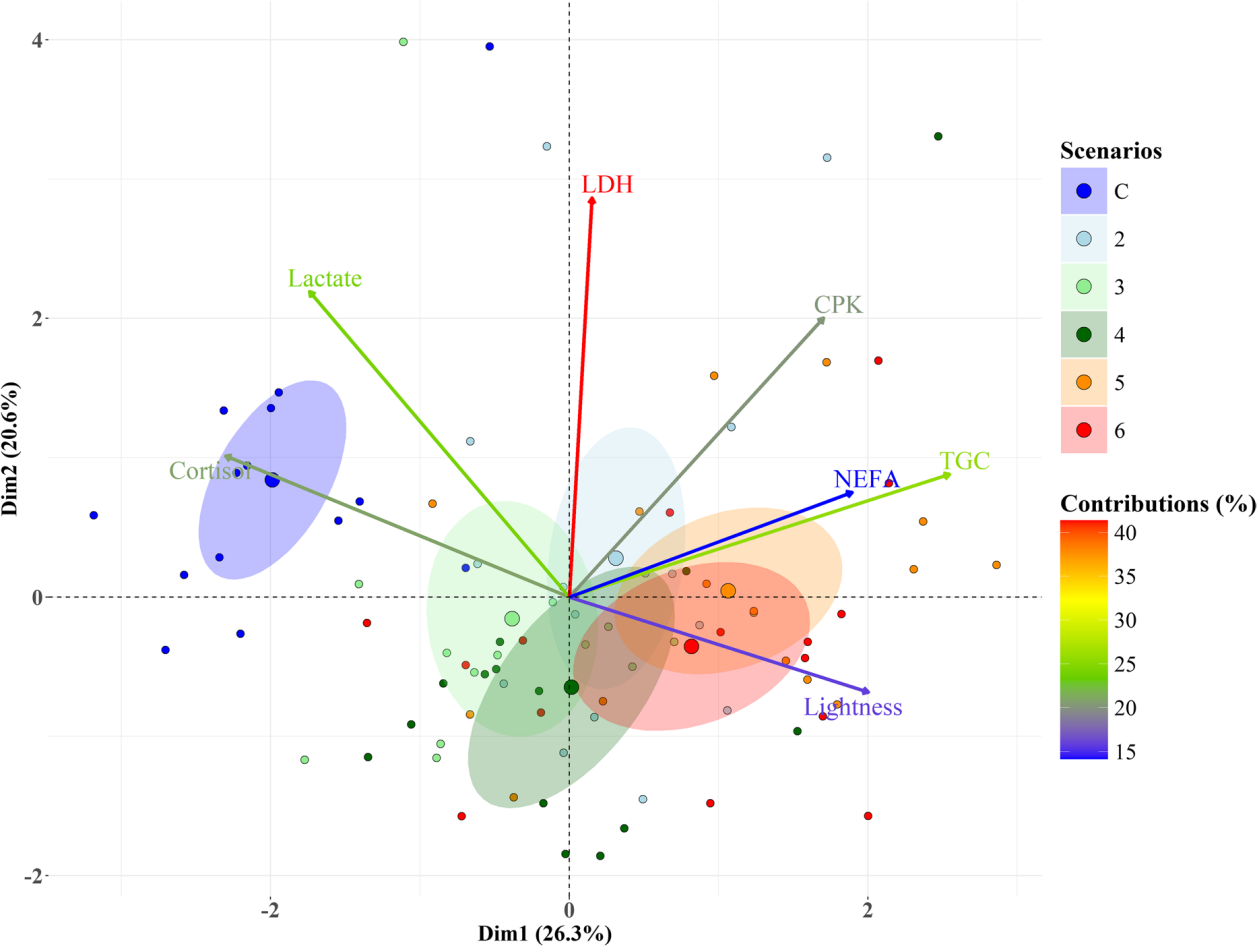
PCA biplot on physiological variables (arrows) measured from trout (dots) subjected to the six hydrological scenarios (colour of the dots). Dim1 and Dim2 represent the first two components. Coloured ellipses group the 20% of the trout equidistant to the centroid from each scenario. The length of the arrows indicates the contribution of the physiological variables to the data variability across the principal components, with percentage contributions shown using a colour gradient. The gradient for lactate, LDH, and CPK corresponds to their contribution to the second dimension, while the remaining variables indicate contributions to the first dimension

In the PCA on physiological variables (as arrows in the Fig. 9) measured from trout (as dots in the Fig. 9) subjected to the six hydrological scenarios, the first two principal components explained virtually half of the variability in the data. The first component accounted for 26.3% of the total variability, while the second component explained 20.6% (Fig. 9). TGC, cortisol, lightness, and NEFA contributed significantly to the first component, with contributions of 25.6%, 20.9%, 15.8%, and 14.2%, respectively (arrow length in Fig. 9). LDH, lactate, and CPK were the major contributors to the second component, with contributions of 41.3%, 24.2%, and 20.2%, respectively (arrow length in Fig. 9). The PCA highlighted a strong positive correlation between NEFA and TGC and a negative correlation between lightness and cortisol (Fig. 9).

The overlap of the hydrological scenario to which the trout were subjected (dot colouration in Fig. 9) onto the PCA biplot revealed a clear segregation of trout according to the six scenarios. The intensity of hydropeaking increased prominently from left to right along the first component and more subtly from top to bottom along the second component (Fig. 9). Intermediate scenarios (2, 3, and 4) were located centrally in the biplot (Fig. 9). Along the first component, cortisol increased towards the left, while lightness and NEFA increased towards the right (arrow directions in Fig. 9), indicating higher cortisol levels but lower lightness and NEFA in the control scenario and less intense hydropeaking scenarios (Fig. 9). Along the second component, LDH, lactate, and CPK levels increased towards the upper part, indicating higher values in the control scenario and lower hydropeaking intensities (Fig. 9).

#### Generalised linear models

The best-performing models were those for lactate (psR^2^ = 0.48; AIC = 564) and cortisol (psR^2^ = 0.39; AIC = 512), followed by models for TGC (psR^2^ = 0.26; AIC = 825) and lightness (psR^2^ = 0.17; AIC = 594). The lactate model provided the best fit, closely followed by the cortisol model (both showed the lowest AICs and highest pseudo R^2^; Table 7). In contrast, the models for LDH and CPK demonstrated the poorest fit (both showed the highest AICs and lowest pseudo R^2^; Table 6). Inundation frequency/duration, rate of change, and level were significant predictors in the cortisol model, and all three of them similarly, inversely, influenced its concentrations (Table 7). Although rate of change and velocity were significant predictors in the lactate model, this metabolite was mainly, inversely determined by the latter (Table 7). Frequency/duration of the inundation and rate of change were significant predictors in the TGC model, but it was the rate of change that had the strongest and positive effect on this metabolite (Table 7). Like lactate, velocity was a significant predictor for lightness but, on the contrary, lightness increased with higher velocities (Table 7).

**Table 7:**
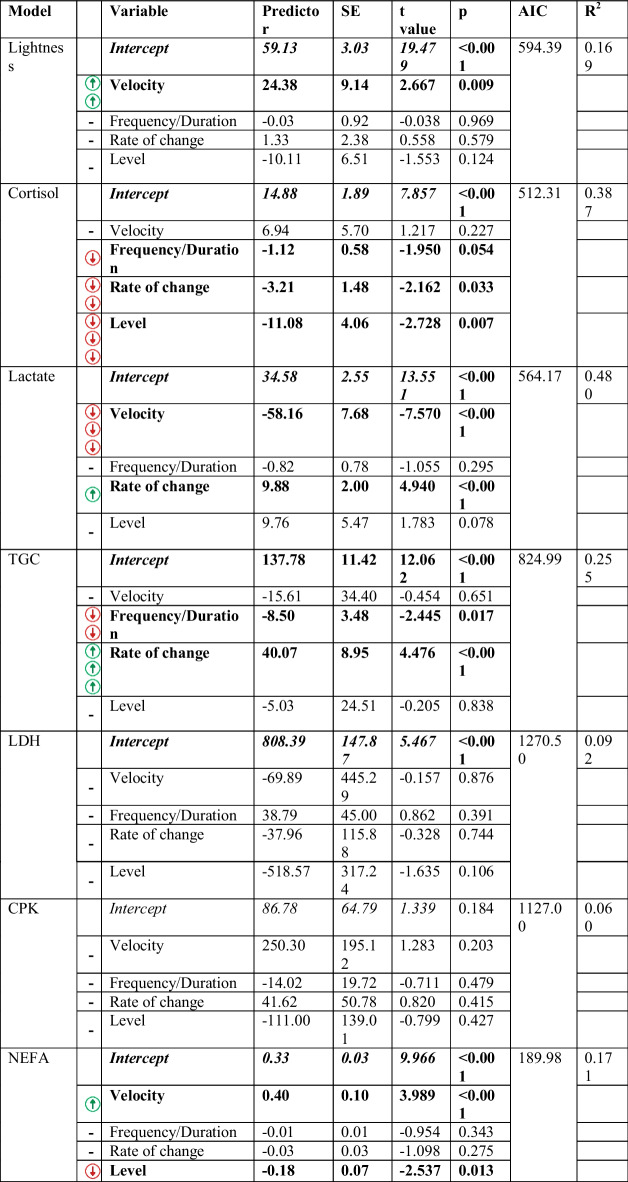
Results of the GLMs for the physiological variables (response). The significant estimators (*p* < 0.05) are marked in bold. The Akaike information criterion (AIC) indicates the goodness of fit of the models for comparison (the lower the AIC, the better the model). The *t*-statistic (*t* value) and pseudo-*R*^*2*^ are also shown. Colour arrows help understand the direction and magnitude of the influence of significant hydrological/hydraulic variables: red-downward arrow, negative influence; green-upward arrow, positive influence; many arrows, strong influence; few arrows, weak influence

## Discussion

This study evidences how hydropeaking imbalances metabolite plasma concentrations in rainbow trout, likely deteriorating the individuals’ and population health (Yada and Tor 2016) and ultimately affecting fish communities (Schultz et al. 2014). We add to previous studies on cortisol (Flodmark et al. 2002; Earley et al. 2019) and explore five other substances, in addition to tissue pigmentation, that literature describes as modified in the face of environmental changes (García-Rejón & Morales 1988). Indeed, our results demonstrate that besides cortisol, lactate and TGC concentrations and tissue lightness can also be used as indicators of rainbow trout stress response due to hydropeaking. Results showed that, while TGC and lightness significantly increased, cortisol and lactate significantly decreased with hydropeaking intensity (Milligan & Wood 1986). The latter two provide a prolonged response signal, which goes beyond the acute stress and towards the chronic stress response in individuals. This prolonged stress response was also identified by Pickering et al. (1991) after 6 to 24 h of exposure in rainbow trouts. Furthermore, our results point to varying relative importance of each of the four hydrological/hydraulic variables characterising hydropeaking depending on the considered response variable. Whereas frequency/duration ratio for the inundation, water level and its rate of change were key in determining cortisol concentrations, velocity was for lactate and lightness, and rate was for TGC. Great variation in concentration of these metabolites affects the HPI axis, which may translate into decrease growth, reproduction, increased toxicant exposure, or disruption of osmoregulation (Mommsen et al. 1999). Consequently, the knowledge on sensitive (physiological) and predictive (hydrological/hydraulic) variables which is provided by these experiments may potentially help in quantifying the impacts of hydropeaking on fish and when defining environmentally friendly operational rules in the power plant, respectively.

Studies focusing on the impact of hydrological disturbance have primarily investigated the mobilisation of lactate and cortisol (Taylor et al. 2012; Faught et al. 2016; De Mercado et al. 2018). Mobilisation of TGC, LDH, CPK, NEFA, and tissue lightness has been widely studied in aquiculture, like indicators of animal welfare (Bermejo-Poza et al. 2017, 2019; Villalba et al. 2024), but their potential mobilisation as indicators of hydropeaking effects remains unexplored. Other metabolites, such as the release of adrenaline, are highly sensitive to certain factors that may not be directly related to a stress response (Reid et al. 1998), while glucose can be used as an indicator of energy reserves or depletion (Clarkson et al. 2005). However, glucose tends to vary in correlation with lactate and cortisol (Ellis et al., 2012) and is closely related to the field of nutrition studies or fasting (Bermejo-Poza et al. 2019). In our study, cortisol and lactate levels were significantly influenced by hydropeaking, but, surprisingly, they tended to decrease with the intensity of the disturbance, instead of increasing as reported in previous studies on other environmental stressors (Costa et al. 2017). For instance, Pickering and Pottinger (1989) and Flodmark et al. (2002) found peak cortisol levels after 30 and 120 min of exposure to a fluctuating flow regime in an artificial stream, respectively, and almost undetectable levels after 24 h, suggesting that the release of cortisol and lactate was closely linked to an acute stress response. Given that hydropeaking lasts longer than 24 h (Bain et al. 1988; Moog 1993; Bejarano et al. 2017), it is likely that the individuals exhausted their cortisol reserves, leading to the release of other products associated with prolonged or even chronic stress (Pérez-Casanova et al. 2008; Yada and Tor 2016). Given the observed responses, it is important to emphasise that cortisol is not a reliable metabolite for indicating prolonged stress (Aerts et al. 2015). Indeed, previous studies have shown that cortisol levels return to baseline after 24 h (McKinley et al. 2025), suggesting that its use as a biomarker is limited to acute stress responses. Therefore, further analyses should incorporate other metabolic products that become relevant once the acute stress pathways are exhausted, particularly those linked to the HPI axis and reflected in secondary responses, such as immune system variation (Moccia et al. 2020), as well as tertiary responses, including growth and behaviour (Moccia et al. 2020). Moreover, extending the experimental period beyond 24 h would be necessary to fully capture potential processes of stress chronification.

Lightness is the histological pigmentation parameter that responded most significantly to hydropeaking. According to studies on animal welfare in aquaculture (Bermejo-Poza et al. 2017, 2019), lightness decreases with acute stress, resulting in a loss of pigmentation and the acquisition of a darker coloration. However, in our study, lightness increased significantly with hydropeaking, leading to a paler coloration in the trout. Villalba et al. (2024) observed that fish exposed to a stressor initially darken (Höglund et al. 2000), but after a relatively long period of exposure, they became paler. This suggests that 24 h of exposure to hydropeaking is sufficient to prolong the stress response in trout beyond the primary (acute) phase, as reflected by the increase in lightness. This pattern, together with the decrease in cortisol levels, is consistent with the initiation of secondary or tertiary stress responses that could, if sustained, lead to a chronic response. Other studies have already highlighted the relationship between chromatic changes and hormonal alterations induced by the stress response (Pavlidis et al. 2006; Vissio et al. 2021). Our findings on lightness agree with those for metabolites measured in plasma, hence demonstrating that histological pigmentation via lightness is a simple and non-invasive method for quantifying the stress levels in rainbow trout individuals exposed to hydropeaking.

Variations in flow velocity and rate of change have been already demonstrated as variables associated with hydropeaking that strongly influence the levels of physiological parameters related to the stress response including cortisol, lactate, or glucose (Nadeau et al. 2010; Costa et al. 2017). However, whereas those studies generally reported upward trends consistent with acute stress responses, the present results reveal the opposite pattern, with decreasing trends. This suggests that, rather than reflecting an acute stress event, the observations indicate metabolite depletion, which subsequently leads to the activation of secondary and tertiary stress responses. Our results agree with these studies but also add other variables such as the frequency and duration of the inundations and the level of the water in determining cortisol concentrations. We go further and, based on our findings, we identify some preliminary reference thresholds for these hydrological/hydraulic variables that could potentially be harmful to rainbow trout individuals. Of course, it would be desirable to expand the experimental range for these variables in future tests. For instance, cortisol levels progressively decreased as the fall rate increased from 0 to 1.2 m h^−1^, while TGC levels increased significantly only when this rate exceeded 0.13 m h^−1^. This interpretation is consistent with the known inhibitory relationship between the two axes: when cortisol decreases, TGC tends to reactivate, leading to higher levels. Such a pattern reflects a transition from a catabolic stress state to an anabolic state, more compatible with recovery and growth. Cortisol also responded to increases in the frequency of inundations, decreasing significantly after one event per day. High flow velocities were primarily associated with decreases in lactate levels, which were significant even with minimal increases in speed above 0.1 m s^−1^. Lightness, on the other hand, exhibited a tendency to increase at velocities exceeding 0.1 m s^−1^.

It is known that the mobilisation of metabolites increases the risk of fish contracting bacterial or fungal diseases (Pickering & Pottinger 1989). However, difficulties may arise not only at the individual level but also at the population level. Kostyniuk et al. (2018) observed that rainbow trout individuals with high TGC levels are dominant in their populations. This adaptation to a highly impacted environment could, in the long term, affect the viability of offspring and the overall quality of the population (Hale & Swearer 2017).

But besides hydrological/hydraulic parameters, the phenomenon of hydropeaking also affects water quality (Bipa et al. 2024). For example, the literature reports sudden temperature fluctuations downstream from large hydropower dams (Béjar et al. 2018; Feng et al. 2018), referred to as *thermopeaking* (Zolezzi et al. 2011). This is due to the contrast between the warmer water from the surface layers of the stratified reservoir (Winton et al. 2019) and the colder water from the river into which the water is discharged. However, the small volume of water managed in the Greenchannel makes thermopeaking unlikely here. The fact that temperature decreased with the intensity of hydropeaking could be more associated with the drop in air temperatures during the experimental period in autumn and winter (ranging from 7 to 16 °C; AEMET 2024) and with the temperature of the water source used (well water at 8 °C). There is limited information in the literature regarding dissolved oxygen variations in rivers subjected to hydropeaking (Pulg et al. 2016; Hayes et al. 2023). Unfortunately, our results cannot shed more light on this aspect, as the observed independence of this water quality variable from hydropeaking could be explained by the constant oxygen supply provided by the oxygenators placed in the mesocosm.

Unlike temperature and dissolved oxygen, variations in water turbidity in our study can be more confidently attributed to the intensity of hydropeaking and its associated hydrological/hydraulic variables. The fact that turbidity levels increased significantly with hydropeaking intensity as well as with water velocity, rate of change, and frequency/duration of inundations may be associated with sediment release from the riverbanks, as other authors have also mentioned (Béjar et al. 2018; Vericat et al. 2020). It is important to highlight, however, that the extent of sediment erosion from the banks (and bed) largely depends on the geology and soil composition (Chakrapani 2005), as well as the presence or absence of vegetation (Asaeda & Rashid 2012). Other studies have also attributed turbidity to sediments released from reservoirs (Greimel et al. 2018), but this would not be the case here.

Poor water quality leads to stress in fish, as noted by Colson et al. (2019). According to the reference values outlined in the regulations (Real Decreto 817/2015) and the guide for the ecological status assessment of surface and groundwater by the Spanish Ministry for Ecological Transition and Demographic Challenge (MITECO 2021), the results obtained in our analysed water samples indicate that the simulated conditions resemble those of a river in very good ecological status. Ammonium, phosphate, nitrate, and pH levels were all within the thresholds for very good ecological status, and dissolved oxygen levels were well above the limits suggesting excellent water quality conditions for the development of individuals (9–16 mg (O_2_)/l; Ritola et al. 1999). In our study, a stress response derived from water temperatures (ranging from 8 to 14.5 °C) is not expected, as this range has not been associated with stress responses in previous studies between 8 and 16 °C (Pérez-Casanova et al. 2008). Turbidity levels can affect plasma cortisol levels in rainbow trout within 24 h when exposed to high concentrations of sediments above 2–3 g l^−1^ (Redding et al. 1987). However, in our study, the maximum nephelometric turbidity levels (15.72 NTU) correspond to a sediment concentration of only 0.005 g l^−1^, which is well below the threshold concentrations reported to cause significant cortisol elevation. Therefore, it is unlikely that turbidity levels in our study had any substantial negative effect on cortisol levels in the organisms. These findings suggest that the impact of hydropeaking on water quality in our study was likely not strong enough to significantly affect the measured physiological stress responses of the individuals. Since this issue is beyond the scope of this work, the effect of hydropeaking on fish through its impact on water quality should be thoroughly investigated in further research.

Hydropeaking can be mitigated in various ways, including both structural and operational measures (Bipa et al. 2024). Our study, through the developed models (e.g. for lactate and cortisol, or lightness), provides valuable information for the application of operational measures aimed at achieving hydropeaking that is compatible with the sustainability of fish communities (*ecopeaking*; Jones 2014). This research identifies the most impactful hydrological/hydraulic variables, along with their thresholds for rainbow trout (velocity 0.1 m s^−1^ and rate of change 0.13 m h^−1^), which is crucial for the sustainable management of hydroelectric power plants, thus helping to restore trout-bearing rivers affected by hydropeaking (Hale & Swearer 2017; Hayes et al. 2023). In addition to these operational measures and considering the other impacts generated in the ecosystem downstream of large dams (Poff et al. 1997; Greimel et al. 2018), other structural mitigation techniques, such as those discussed by Moreira et al. (2019), could be applied. These would help create areas free from abrupt and frequent fluctuations in water level changes and/or areas with below-average flow velocities. These “buffer” areas of hydropeaking would not only prevent the stranding of individuals or loss of their eggs (Greimel et al. 2018; Hayes et al. 2024), but also, they would guarantee the maintenance of normal levels of stress metabolites in individuals, avoiding the proliferation of diseases and mortality. These benefits ultimately would lead to a good status of the fish community and an improved functioning of a river reach, and, consequently, its ecological restoration.

Studies have highlighted the ability of fish to refuge in certain areas of the river where environmental conditions are not significantly altered by hydropeaking, such as in the shelter of vegetation or large rocks and boulders (Baladrón et al. 2021). This could result in heterogeneous measurements for the physiological stress response to hydropeaking along river reaches in nature. Additionally, power plants limit the range of hydropeaking scenarios tests, which ideally should be wide. One of the advantages of the Greenchannel, as evidenced in our study, is that hydrological scenarios can be simulated on demand, and that environmental conditions characterising the scenarios are to some extent homogeneously reached throughout the whole experimental area ensuring that the stress responses measured in all individuals can be directly attributed to them. Despite the careful experimental design to ensure homogeneity within the channel, slight velocity losses due to friction were observed in specific points of the lower sections likely becoming shelter areas for fish. Although these sections represent a very low percentage of the total experimental area, future experiments would benefit from the installation of nets to keep fish away, while taking care of the resulting alteration of the hydraulic conditions. In any case, mesocosms like the Greenchannel are shown as useful facilities where easily and accurately test both operational and structural restoration measures. Particularly, this study demonstrates the potentiality of this kind of facilities in exploring hydropeaking, such as implementing and monitoring the effectiveness of hydropeaking impacts restoration techniques.

## Conclusion

This study demonstrates how hydroelectric power production in large plants can pose a risk to the health of salmonids, such as rainbow trout. Water velocity and rate of change, as well as inundation frequency, duration, and water level, all of which are altered in rivers affected by hydropeaking (Bejarano et al. 2017), have led to significant changes in physiological variables (measured in plasma and tissue pigmentation) indicative of stress responses in individuals. Among the physiological variables studied, cortisol, lactate, and triglycerides were significantly affected by hydropeaking, as well as tissue lightness, which stood out for being a non-invasive variable. The mobilisation of metabolites in trout individuals suggests a prolonged stress response under hydropeaking scenarios of 24 h, although demonstrating true chronification would require longer exposure times, and the assessment of additional indicators, such as growth and behaviour. Furthermore, the results of this study show an increase in water turbidity with hydropeaking, with potential negative impacts on trout. Based on our findings, this study proposes the restoration of river sections downstream of large hydroelectric plants through a combination of operational techniques and the creation of buffer zones that maintain the most influential hydrological and hydraulic parameters below the defined thresholds, according to the response variables analysed.

## Supplementary Information

Below is the link to the electronic supplementary material.

ESM 1 (DOCX 3.49 MB)

## Data Availability

Data are available on request to the corresponding author.
