# Supplementary material for: Physiological stress response to hydropeaking in rainbow trout (Oncorhynchus mykiss)
Source: Fish Physiol Biochem. 2026 Jan 16;52(1):15. doi: 10.1007/s10695-026-01633-z (PMC12811275; doi:10.1007/s10695-026-01633-z)
Supplement: Supplementary file 1 — (DOCX 3.49 MB) [file 10695_2026_1633_MOESM1_ESM.docx]

**Physiological stress in rainbow trout affected by hydropeaking**

Raul Hernandez-Marchena^1^, Álvaro De la Llave-Propín^2^, Joaquín Solana-Gutierrez^1^, *Maria Dolores Bejarano^1^

^1^Department of Systems and Natural Resources, Universidad Politécnica de Madrid, 28040, Madrid, Spain

^2^Departament of Agrarian Production, Universidad Politécnica de Madrid, 28040, Madrid, Spain

*Corresponding autor: mariadolores.bejarano@upm.es

**Supplementary material**

**
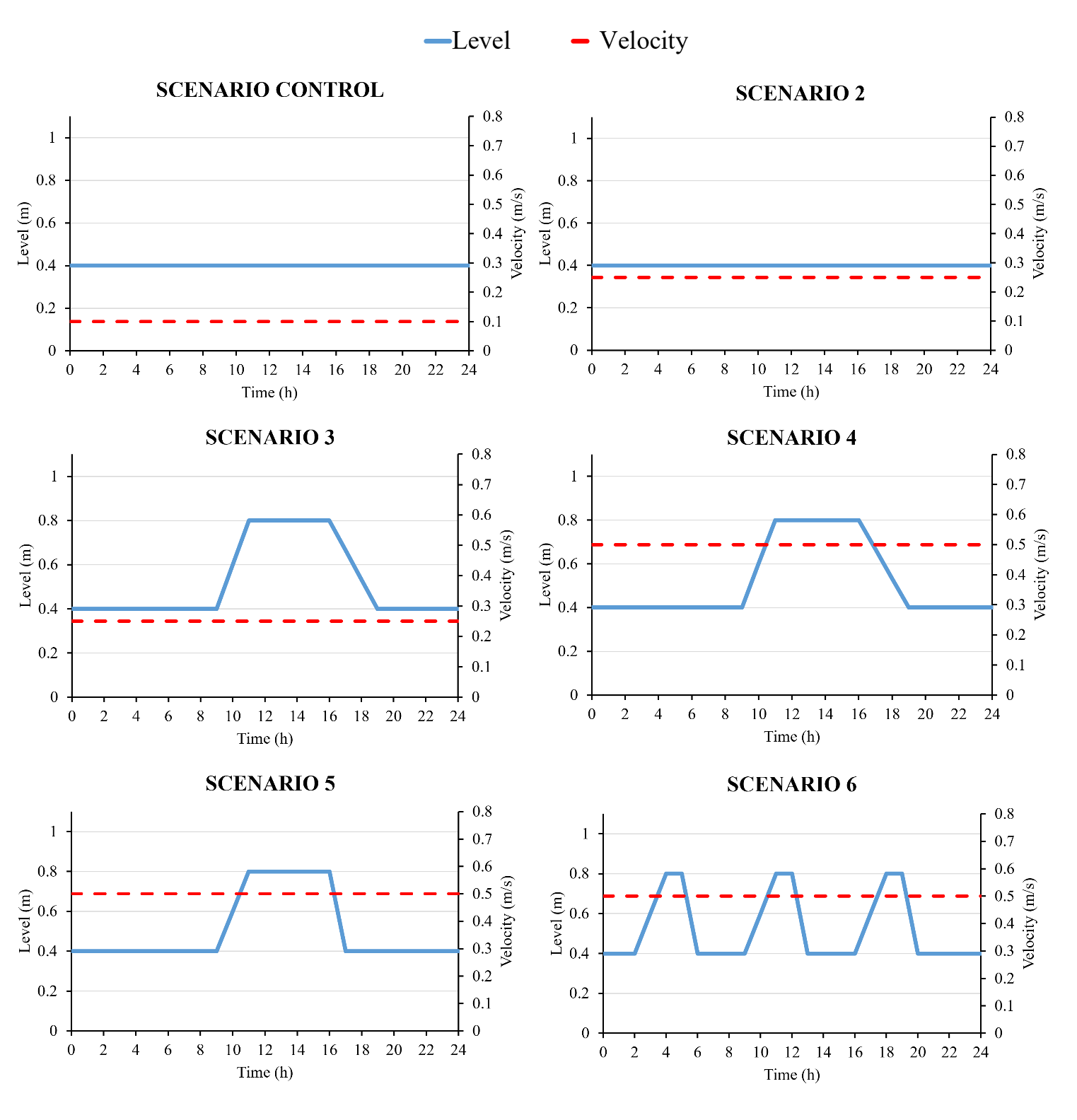
**

**Figure S.1.** Graphs representing the different experimental scenarios. The water level is indicated by a solid blue line, while the velocity is represented by a dashed red line.

**
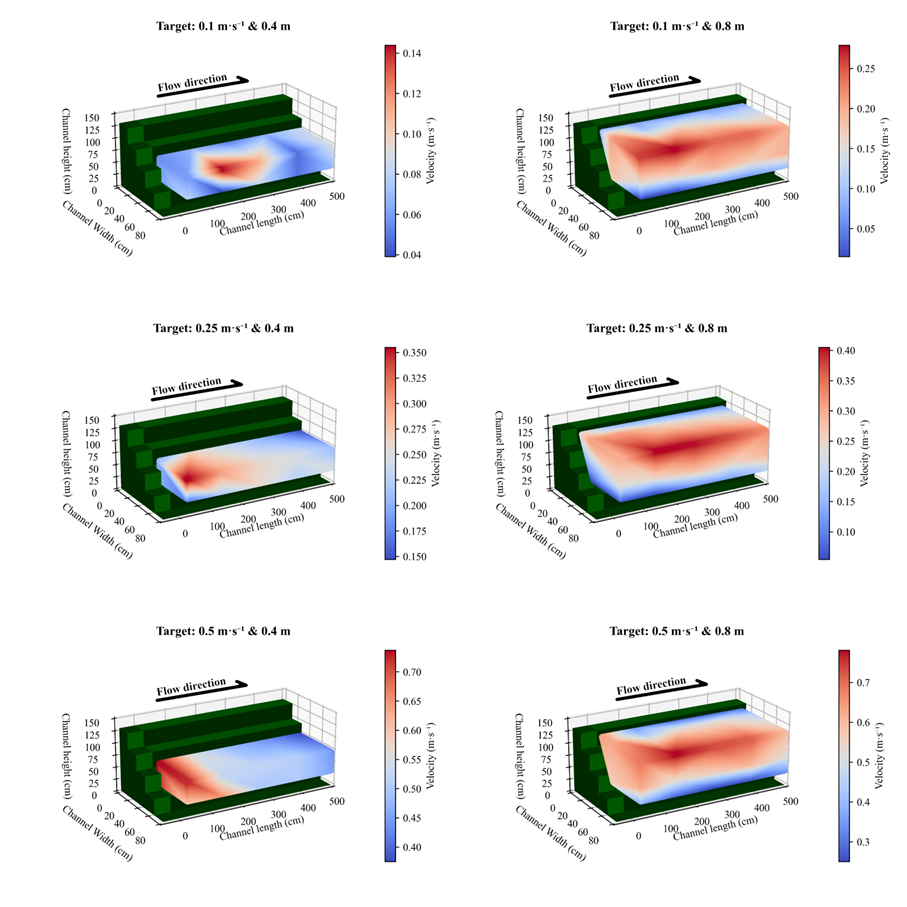
**

**Figure S.2.** Velocity mapping under various setpoints. The figures on the left depict setpoints at water level of 0.4 m with velocities of 0.1, 0.25, and 0.5 m s^-1^, from top to bottom. On the right, the setpoints are shown for a water level of 0.8 m with velocities of 0.1, 0.25, and 0.5 m s^-1^, also from top to bottom.


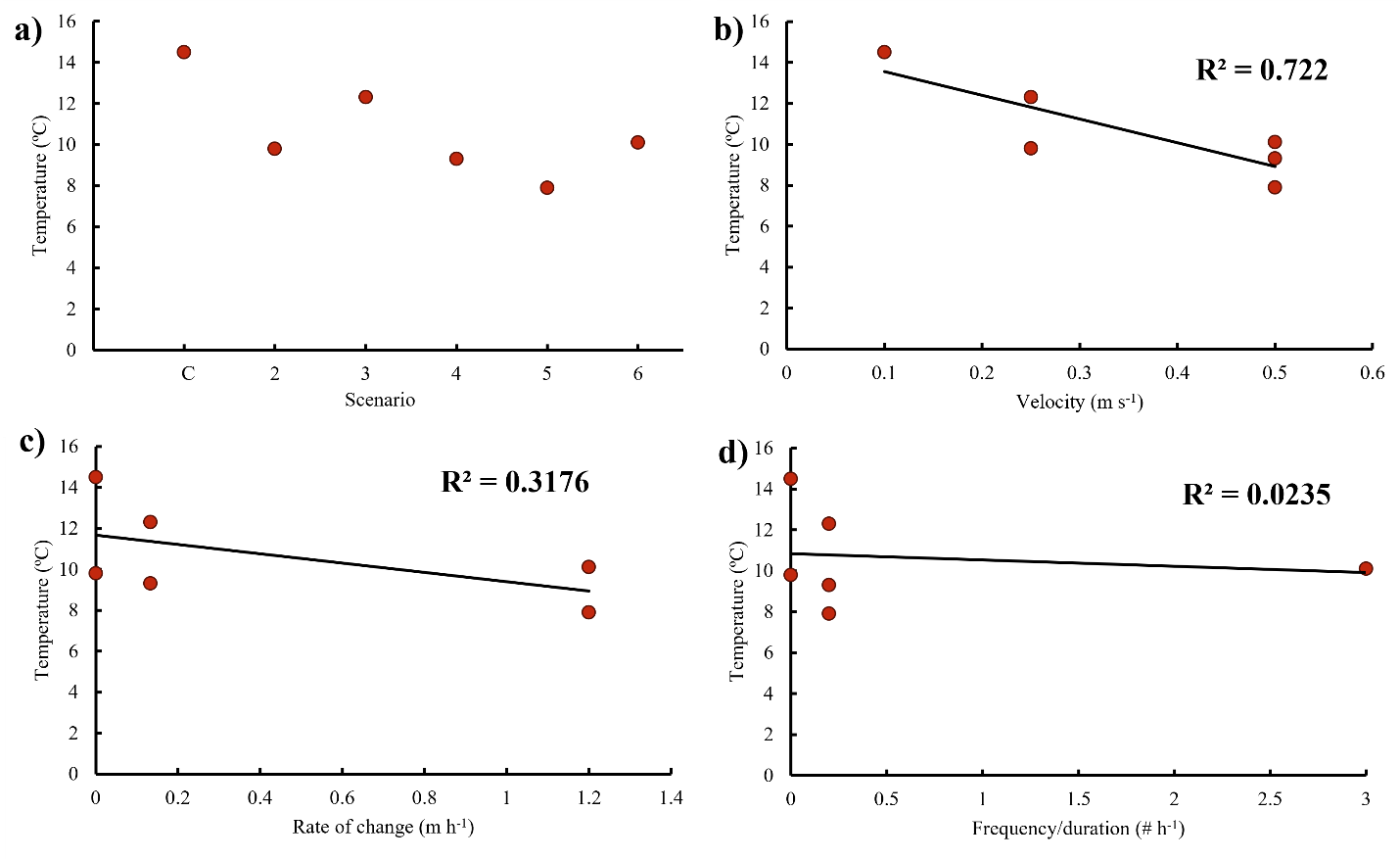


**Figure S.3.** Temperature variation with hydrological scenarios (a), water velocities (b), decent rates (c), and frequency/duration (d). R^2^ denotes Pearson’s correlation coefficient.


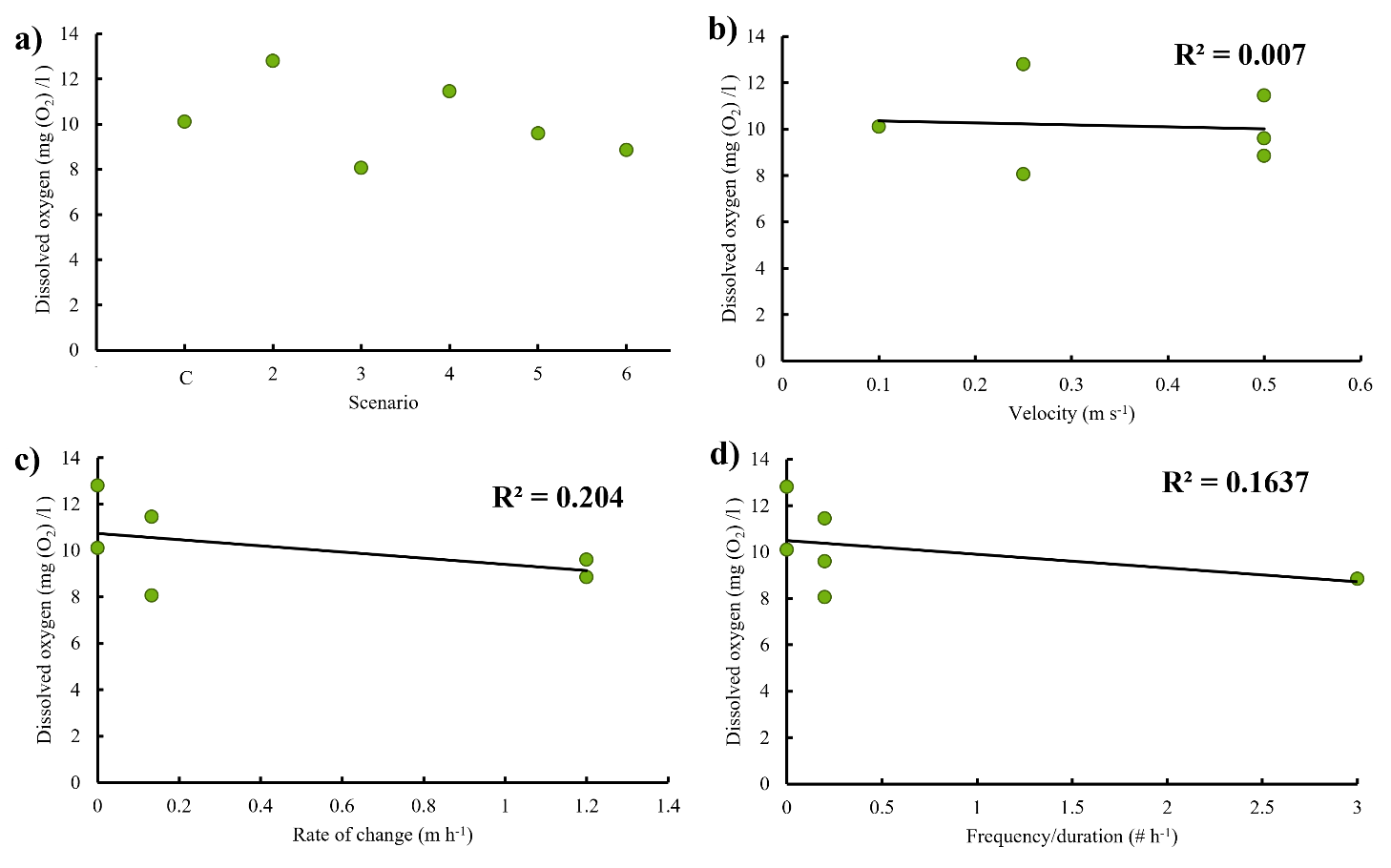


**Figure S.4.** Variation of dissolved oxygen with hydrological scenarios (a), water velocities (b), decent rates (c), and frequency/duration (d). R^2^ denotes Pearson’s correlation coefficient.


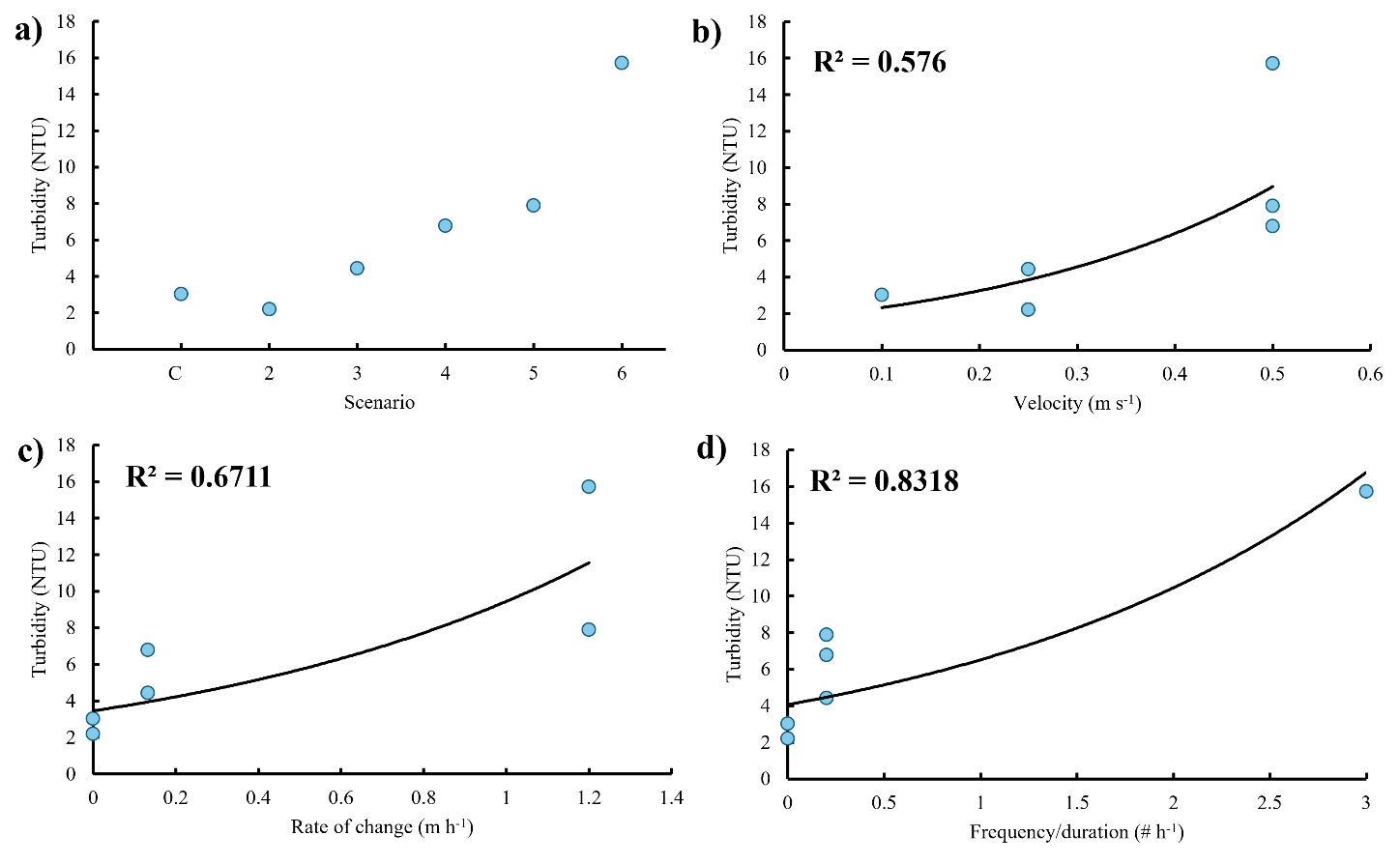


**Figure S.5.** Variation of turbidity with hydrological scenarios (a), water velocities (b), decent rates (c), and frequency/duration (d). R^2^ denotes Pearson’s correlation coefficient.

**Table S.1.** Histological pigmentation variables obtained for each scenario (Sc.), for each individual (Ind.), with three measurements taken for each parameter. The weight (Weig.) and length (Len.) of each trout are also included. L, lightness; a*, red; b*, yellow. Chroma (C* = (a^2^ + b^2^)^1/2^) and hue (h* = arctan(b*/a*) × 57.29) values were calculated in this method. The subscripts indicate the measurement number. In scenarios 1 and 5, only two measurements were taken, with the third measurement indicated by a dash (-).

| **Ind.** | **Sc.** | **Weig.** | **Len.** | **L_1_** | **L_2_** | **L_3_** | **h*_1_** | **h*_2_** | **h*_3_** | **C*_1_** | **C*_2_** | **C*_3_** | **a*_1_** | **a*_2_** | **a*_3_** | **b*_1_** | **b*_2_** | **b*_3_** |
| --- | --- | --- | --- | --- | --- | --- | --- | --- | --- | --- | --- | --- | --- | --- | --- | --- | --- | --- |
| 1 | 1 | 263 | 28.5 | 62.12 | 60.32 | - | 20.70 | 47.09 | - | 4.46 | 7.73 | - | 4.17 | 5.27 | - | 1.58 | 5.66 | - |
| 2 | 1 | 270 | 28.0 | 61.30 | 51.57 | - | 298.08 | 75.63 | - | 1.92 | 1.32 | - | 0.9 | 0.33 | - | -1.69 | 1.28 | - |
| 3 | 1 | 268 | 28.5 | 60.08 | 58.97 | - | 347.81 | 350.45 | - | 1.11 | 2.07 | - | 1.09 | 2.04 | - | -0.23 | -0.34 | - |
| 4 | 1 | 236 | 27.8 | 46.93 | 50.71 | - | 59.09 | 47.21 | - | 7.13 | 3.11 | - | 3.66 | 2.11 | - | 6.11 | 2.28 | - |
| 5 | 1 | 273 | 28.5 | 38.61 | 35.29 | - | 85.60 | 56.66 | - | 8.26 | 2.67 | - | 0.63 | 1.47 | - | 8.24 | 2.23 | - |
| 6 | 1 | 270 | 28.5 | 45.00 | 44.58 | - | 70.47 | 67.57 | - | 11.18 | 9.57 | - | 3.74 | 3.65 | - | 10.54 | 8.84 | - |
| 7 | 1 | 250 | 28.5 | 59.98 | 63.22 | - | 328.47 | 331.27 | - | 2.49 | 4.03 | - | 2.12 | 3.53 | - | -1.30 | -1.94 | - |
| 8 | 1 | 242 | 27.2 | 53.70 | 55.42 | - | 69.93 | 64.39 | - | 6.38 | 3.42 | - | 2.19 | 1.48 | - | 5.99 | 3.09 | - |
| 9 | 1 | 242 | 27.4 | 58.68 | 53.31 | - | 16.94 | 50.63 | - | 3.92 | 5.38 | - | 3.75 | 3.41 | - | 1.14 | 4.16 | - |
| 10 | 1 | 271 | 29.5 | 55.09 | 46.66 | - | 27.06 | 61.29 | - | 2.91 | 7.05 | - | 2.59 | 3.39 | - | 1.32 | 6.19 | - |
| 11 | 1 | 280 | 28.3 | 67.22 | 52.39 | - | 63.85 | 60.41 | - | 15.22 | 4.36 | - | 6.71 | 2.15 | - | 13.66 | 3.79 | - |
| 12 | 1 | 261 | 28.0 | 53.33 | 60.11 | - | 77.64 | 15.86 | - | 4.44 | 3.09 | - | 0.95 | 2.98 | - | 4.34 | 0.85 | - |
| 13 | 1 | 240 | 27.7 | 58.26 | 44.28 | - | 347.04 | 70.80 | - | 2.98 | 5.45 | - | 2.91 | 1.79 | - | -0.67 | 5.15 | - |
| 14 | 1 | 278 | 29.0 | 55.25 | 60.14 | - | 7.90 | 358.37 | - | 3.01 | 3.97 | - | 2.99 | 3.97 | - | 0.41 | -0.11 | - |
| 15 | 1 | 234 | 27.5 | 59.06 | 52.40 | - | 15.45 | 345.77 | - | 5.20 | 3.02 | - | 5.01 | 2.93 | - | 1.39 | -0.74 | - |
| 16 | 2 | 362 | 31.6 | 69.11 | 71.63 | 51.19 | 281.93 | 321.72 | 57.64 | 2.32 | 2.73 | 3.39 | 0.48 | 2.14 | 1.81 | -2.27 | -1.69 | 2.86 |
| 17 | 2 | 330 | 31.5 | 62.57 | 62.37 | 61.71 | 358.35 | 17.36 | 26.96 | 2.77 | 1.83 | 2.80 | 2.77 | 1.75 | 2.49 | -0.08 | 0.55 | 1.27 |
| 18 | 2 | 191 | 25.5 | 67.48 | 75.16 | 73.69 | 182.12 | 245.08 | 263.44 | 0.23 | 1.31 | 2.71 | -0.23 | -0.55 | -0.31 | -0.01 | -1.18 | -2.70 |
| 19 | 2 | 300 | 30.0 | 70.45 | 70.86 | 69.76 | 273.80 | 273.56 | 270.97 | 3.99 | 2.29 | 2.83 | 0.26 | 0.14 | 0.05 | -3.98 | -2.28 | -2.83 |
| 20 | 2 | 274 | 29.5 | 64.39 | 63.23 | 60.13 | 323.90 | 2.07 | 4.56 | 4.42 | 3.15 | 4.95 | 3.57 | 3.15 | 4.93 | 0.11 | 0.11 | 0.39 |
| 21 | 2 | 345 | 30.5 | 54.58 | 58.21 | 64.22 | 265.79 | 210.83 | 304.91 | 1.32 | 1.15 | 3.00 | -0.1 | -0.99 | 1.72 | -1.32 | -0.59 | -2.46 |
| 22 | 2 | 303 | 29.5 | 66.79 | 65.89 | 61.55 | 108.41 | 120.50 | 59.79 | 4.20 | 3.90 | 4.32 | -1.33 | -1.98 | 2.17 | 3.99 | 3.36 | 3.73 |
| 23 | 2 | 317 | 30.0 | 62.38 | 63.02 | 59.87 | 113.83 | 93.00 | 60.42 | 3.41 | 2.29 | 1.14 | -1.38 | -0.12 | 0.56 | 3.11 | 2.29 | 0.99 |
| 24 | 2 | 341 | 31.5 | 70.71 | 63.36 | 67.18 | 293.69 | 300.65 | 298.55 | 6.30 | 2.51 | 4.40 | 2.53 | 1.28 | 2.10 | -5.77 | -2.16 | -3.86 |
| 25 | 2 | 268 | 29.6 | 61.10 | 69.69 | 72.60 | 219.47 | 294.15 | 290.11 | 1.08 | 4.44 | 3.64 | -0.83 | 1.82 | 1.25 | -0.68 | -4.06 | -3.42 |
| 26 | 2 | 419 | 32.5 | 56.45 | 57.56 | 50.08 | 93.78 | 67.10 | 81.28 | 1.80 | 1.56 | 5.70 | -0.12 | 0.61 | 0.86 | 1.79 | 1.43 | 5.64 |
| 27 | 2 | 303 | 30.0 | 67.11 | 72.72 | 70.41 | 148.21 | 271.99 | 115.26 | 0.30 | 1.63 | 1.39 | -0.26 | 0.06 | -0.59 | 0.16 | -1.63 | 1.26 |
| 28 | 2 | 345 | 31.4 | 52.90 | 60.61 | 50.07 | 59.64 | 55.70 | 73.08 | 5.13 | 2.60 | 3.78 | 2.59 | 1.47 | 1.10 | 4.43 | 2.15 | 3.61 |
| 29 | 2 | 283 | 29.4 | 61.77 | 63.75 | 64.49 | 302.39 | 210.35 | 189.74 | 1.13 | 1.38 | 0.41 | 0.61 | -1.19 | -0.40 | -0.95 | -0.70 | -0.07 |
| 30 | 2 | 271 | 28.3 | 67.28 | 65.44 | 63.72 | 278.66 | 286.52 | 300.40 | 4.16 | 3.63 | 3.34 | 0.63 | 1.03 | 1.69 | -4.11 | -3.48 | -2.88 |
| 31 | 3 | 314 | 30.0 | 54.47 | 60.37 | 44.54 | 76.38 | 63.32 | 88.02 | 11.08 | 6.34 | 10.38 | 2.61 | 2.85 | 0.36 | 10.76 | 5.66 | 10.37 |
| 32 | 3 | 272 | 28.6 | 59.36 | 60.61 | 61.79 | 67.97 | 17.28 | 353.59 | 7.71 | 3.03 | 5.66 | 2.89 | 2.89 | 5.62 | 7.14 | 0.90 | -0.63 |
| 33 | 3 | 270 | 29.0 | 61.59 | 62.36 | 62.23 | 66.62 | 61.07 | 63.77 | 8.44 | 6.70 | 6.62 | 3.35 | 3.24 | 2.93 | 7.74 | 5.86 | 5.94 |
| 34 | 3 | 247 | 27.5 | 64.60 | 65.29 | 68.07 | 310.57 | 305.07 | 309.94 | 3.25 | 2.91 | 3.31 | 2.11 | 1.67 | 2.13 | -2.47 | -2.39 | -2.54 |
| 35 | 3 | 258 | 28.0 | 48.43 | 53.30 | 47.78 | 97.58 | 124.49 | 96.47 | 3.78 | 4.16 | 4.29 | -0.5 | -2.36 | -0.48 | 3.74 | 3.43 | 4.26 |
| 36 | 3 | 263 | 28.0 | 50.96 | 50.83 | 55.95 | 49.98 | 55.56 | 25.67 | 5.05 | 6.32 | 2.65 | 3.24 | 3.57 | 2.39 | 3.86 | 5.21 | 1.15 |
| 37 | 3 | 248 | 27.5 | 53.16 | 53.90 | 58.09 | 42.13 | 23.74 | 12.36 | 2.87 | 2.37 | 4.14 | 2.13 | 2.17 | 4.05 | 1.92 | 0.96 | 0.89 |
| 38 | 3 | 205 | 27.5 | 54.73 | 62.84 | 67.15 | 14.84 | 296.5 | 284.71 | 1.46 | 2.86 | 6.46 | 1.42 | 1.28 | 1.64 | 0.38 | -2.56 | -6.25 |
| 39 | 3 | 297 | 29.5 | 62.33 | 59.39 | 65.00 | 69.53 | 62.62 | 81.70 | 8.61 | 5.77 | 5.55 | 3.01 | 2.65 | 0.80 | 8.07 | 5.12 | 5.49 |
| 40 | 3 | 293 | 30.0 | 66.97 | 58.60 | 40.50 | 83.57 | 84.08 | 87.31 | 9.37 | 9.67 | 11.97 | 1.05 | 1.00 | 0.56 | 9.31 | 9.62 | 11.96 |
| 41 | 3 | 315 | 29.6 | 69.51 | 64.20 | 65.14 | 36.38 | 56.05 | 69.47 | 4.36 | 3.53 | 6.70 | 3.51 | 1.97 | 2.35 | 2.59 | 2.93 | 6.28 |
| 42 | 3 | 287 | 30.2 | 69.30 | 60.19 | 65.16 | 28.06 | 42.59 | 52.96 | 2.68 | 3.25 | 2.92 | 2.36 | 2.39 | 1.76 | 1.26 | 2.20 | 2.33 |
| 43 | 3 | 236 | 27.0 | 67.79 | 62.02 | 57.51 | 307.64 | 318.1 | 46.77 | 5.11 | 2.74 | 1.67 | 3.12 | 2.04 | 1.14 | -4.05 | -1.83 | 1.22 |
| 44 | 3 | 255 | 28.5 | 64.05 | 64.41 | 60.00 | 337.04 | 35.76 | 75.41 | 1.55 | 1.10 | 1.81 | 1.43 | 0.90 | 0.46 | -0.61 | 0.65 | 1.75 |
| 45 | 3 | 261 | 28.0 | 54.95 | 55.29 | 55.01 | 66.68 | 75.49 | 69.58 | 4.86 | 3.95 | 6.05 | 1.93 | 0.99 | 2.11 | 4.47 | 3.82 | 5.67 |
| 46 | 4 | 387 | 32.5 | 65.99 | 54.38 | 63.60 | 317.98 | 60.77 | 320.38 | 3.79 | 5.12 | 4.90 | 2.81 | 2.50 | 3.77 | -2.54 | 4.47 | -3.12 |
| 47 | 4 | 302 | 30.4 | 69.78 | 71.61 | 66.32 | 248.74 | 353.51 | 332.08 | 1.81 | 0.66 | 0.40 | -0.66 | 0.66 | 0.36 | -1.69 | -0.07 | -0.19 |
| 48 | 4 | 323 | 30.3 | 57.14 | 47.81 | 55.92 | 44.72 | 60.00 | 65.10 | 2.68 | 5.42 | 2.82 | 1.9 | 2.71 | 1.19 | 1.88 | 4.70 | 2.56 |
| 49 | 4 | 335 | 31.4 | 68.43 | 70.88 | 66.39 | 323.04 | 337.49 | 12.67 | 2.03 | 2.24 | 0.88 | 1.62 | 2.07 | 0.86 | -1.22 | -0.86 | 0.19 |
| 50 | 4 | 347 | 31.0 | 64.98 | 69.57 | 65.35 | 308.22 | 309.77 | 264.45 | 3.02 | 2.10 | 0.44 | 1.87 | 1.34 | -0.04 | -2.37 | -1.61 | -0.44 |
| 51 | 4 | 293 | 29.7 | 59.56 | 59.29 | 58.91 | 257.34 | 308.43 | 274.55 | 4.26 | 3.24 | 4.62 | -0.93 | 2.01 | 0.37 | -4.16 | -2.54 | -4.60 |
| 52 | 4 | 318 | 30.8 | 51.31 | 56.70 | 58.25 | 303.87 | 303.14 | 308.82 | 4.56 | 8.57 | 10.50 | 2.54 | 4.69 | 6.58 | -3.78 | -7.18 | -8.18 |
| 53 | 4 | 363 | 32.2 | 56.64 | 59.59 | 57.95 | 280.62 | 297.17 | 307.93 | 4.41 | 4.64 | 7.47 | 0.81 | 2.12 | 4.59 | -4.34 | -4.12 | -5.89 |
| 54 | 4 | 308 | 30.2 | 58.32 | 62.02 | 60.36 | 313.30 | 319.43 | 316.51 | 5.08 | 3.96 | 5.69 | 3.48 | 3.01 | 4.13 | -3.69 | -2.57 | -3.92 |
| 55 | 4 | 366 | 32.3 | 55.41 | 56.64 | 64.49 | 349.22 | 326.99 | 318.14 | 3.23 | 4.29 | 6.54 | 3.17 | 3.60 | 4.87 | -0.60 | -2.34 | -4.36 |
| 56 | 4 | 319 | 30.3 | 59.84 | 61.41 | 66.90 | 4.87 | 159.14 | 46.82 | 0.79 | 0.63 | 0.78 | 0.78 | -0.59 | 0.53 | 0.07 | 0.22 | 0.57 |
| 57 | 4 | 228 | 27.7 | 54.11 | 56.48 | 53.14 | 309.15 | 328.63 | 325.73 | 3.94 | 1.88 | 2.86 | 2.49 | 1.60 | 2.36 | -3.06 | -0.98 | -1.61 |
| 58 | 4 | 290 | 30.5 | 51.55 | 53.87 | 55.97 | 320.48 | 315.9 | 323.78 | 7.24 | 7.67 | 8.14 | 5.59 | 5.51 | 6.57 | -4.61 | -5.34 | -4.81 |
| 59 | 4 | 317 | 30.1 | 66.15 | 64.87 | 65.99 | 274.94 | 272.71 | 287.19 | 4.43 | 3.79 | 3.38 | 0.38 | 0.18 | 1.00 | -4.41 | -3.78 | -3.23 |
| 60 | 4 | 303 | 29.8 | 77.32 | 76.68 | 77.10 | 320.88 | 324.68 | 327.66 | 5.22 | 5.96 | 5.73 | 4.05 | 4.86 | 4.84 | -3.30 | -3.44 | -3.06 |
| 61 | 5 | 259 | 28.4 | 70.15 | 73.43 | - | 59.35 | 312.70 | - | 3.42 | 3.91 | - | 1.74 | 2.65 | - | 2.94 | -2.88 | - |
| 62 | 5 | 293 | 29.0 | 67.56 | 65.81 | - | 338.44 | 358.51 | - | 3.50 | 3.29 | - | 3.25 | 3.29 | - | -1.28 | -0.09 | - |
| 63 | 5 | 244 | 27.3 | 68.36 | 70.87 | - | 20.55 | 359.79 | - | 5.21 | 4.24 | - | 4.88 | 4.24 | - | 1.83 | -0.02 | - |
| 64 | 5 | 316 | 31.0 | 66.28 | 64.57 | - | 13.51 | 46.51 | - | 4.62 | 6.92 | - | 4.49 | 4.76 | - | 1.08 | 5.02 | - |
| 65 | 5 | 274 | 29.0 | 73.52 | 73.47 | - | 315.67 | 322.07 | - | 8.09 | 7.40 | - | 5.78 | 5.83 | - | -5.65 | -4.55 | - |
| 66 | 5 | 257 | 28.5 | 69.13 | 68.24 | - | 294.81 | 270.06 | - | 4.29 | 1.97 | - | 1.8 | 0.00 | - | -3.89 | -1.97 | - |
| 67 | 5 | 260 | 29.3 | 67.90 | 74.01 | - | 343.75 | 328.73 | - | 3.92 | 3.85 | - | 3.76 | 3.29 | - | -1.10 | -2.00 | - |
| 68 | 5 | 244 | 28.0 | 68.77 | 72.01 | - | 262.38 | 274.78 | - | 2.08 | 1.14 | - | -0.28 | 0.09 | - | -2.06 | -1.13 | - |
| 69 | 5 | 214 | 26.7 | 65.08 | 59.23 | 61.37 | 297.17 | 314.72 | 60.93 | 3.96 | 4.11 | 5.17 | 1.81 | 2.89 | 2.51 | -3.53 | -2.92 | 4.52 |
| 70 | 5 | 257 | 28.7 | 65.91 | 65.26 | - | 340.42 | 25.53 | - | 2.72 | 3.03 | - | 2.56 | 2.73 | - | -0.91 | 1.31 | - |
| 71 | 5 | 251 | 28.5 | 63.16 | 67.14 | 55.33 | 11.97 | 323.99 | 81.70 | 3.70 | 5.30 | 11.22 | 3.62 | 4.29 | 1.62 | 0.77 | -3.12 | 11.1 |
| 72 | 5 | 299 | 30.0 | 50.07 | 53.13 | 51.95 | 105.22 | 300.46 | 81.14 | 1.31 | 4.45 | 1.71 | -0.34 | 2.25 | 0.26 | 1.26 | -3.83 | 1.69 |
| 73 | 5 | 328 | 30.7 | 56.18 | 54.65 | 56.71 | 46.18 | 44.93 | 34.63 | 4.01 | 5.87 | 3.52 | 2.78 | 4.16 | 2.89 | 2.89 | 4.15 | 2.00 |
| 74 | 5 | 287 | 28.7 | 62.57 | 60.89 | 55.39 | 49.95 | 38.79 | 68.39 | 4.39 | 3.23 | 5.37 | 2.82 | 2.51 | 1.98 | 3.36 | 2.02 | 5.00 |
| 75 | 5 | 288 | 29.0 | 65.24 | 55.88 | 54.48 | 86.12 | 68.68 | 67.09 | 2.72 | 5.46 | 5.83 | 0.18 | 1.99 | 2.27 | 2.72 | 5.09 | 5.37 |
| 76 | 6 | 205 | 26.5 | 63.44 | 69.13 | 57.83 | 20.73 | 344.18 | 71.43 | 10.07 | 7.91 | 9.74 | 9.42 | 7.61 | 3.10 | 3.56 | -2.16 | 9.24 |
| 77 | 6 | 238 | 26.0 | 81.53 | 74.79 | 68.81 | 13.5 | 7.28 | 41.27 | 5.31 | 6.33 | 5.43 | 5.16 | 6.28 | 4.08 | 1.24 | 0.80 | 3.58 |
| 78 | 6 | 227 | 26.5 | 77.69 | 74.13 | 70.00 | 342.09 | 359.61 | 35.31 | 6.92 | 5.03 | 5.70 | 6.58 | 5.03 | 4.65 | -2.13 | -0.03 | 3.30 |
| 79 | 6 | 205 | 26.5 | 60.39 | 56.47 | 62.94 | 38.94 | 65.75 | 58.95 | 6.83 | 8.07 | 5.95 | 5.31 | 3.31 | 3.07 | 4.29 | 7.36 | 5.10 |
| 80 | 6 | 266 | 28.5 | 67.08 | 66.44 | 60.55 | 308.82 | 305.86 | 19.55 | 4.67 | 4.65 | 1.63 | 2.93 | 2.72 | 1.54 | -3.64 | -3.77 | 0.55 |
| 81 | 6 | 253 | 27.3 | 43.33 | 38.85 | 48.29 | 18.18 | 68.74 | 14.76 | 3.35 | 5.59 | 1.91 | 3.18 | 2.03 | 1.84 | 1.04 | 5.21 | 0.49 |
| 82 | 6 | 223 | 26.6 | 56.18 | 54.67 | 55.41 | 356.52 | 19.56 | 64.75 | 1.54 | 1.83 | 2.63 | 1.54 | 1.72 | 1.12 | -0.09 | 0.61 | 2.38 |
| 83 | 6 | 267 | 29.0 | 80.18 | 78.67 | 67.50 | 334.94 | 350.71 | 56.01 | 4.29 | 4.10 | 5.36 | 3.88 | 4.05 | 3.00 | -1.82 | -0.66 | 4.45 |
| 84 | 6 | 301 | 29.0 | 60.32 | 60.01 | 63.69 | 72.71 | 60.85 | 89.52 | 6.05 | 3.14 | 6.78 | 1.80 | 1.53 | 0.06 | 5.78 | 2.74 | 6.78 |
| 85 | 6 | 248 | 28.6 | 72.06 | 65.99 | 65.59 | 7.01 | 41.00 | 72.44 | 3.70 | 3.97 | 4.57 | 3.67 | 3.00 | 1.38 | 0.45 | 2.61 | 4.36 |
| 86 | 6 | 250 | 28.5 | 65.64 | 65.57 | 63.26 | 61.49 | 77.38 | 63.78 | 4.30 | 6.55 | 5.73 | 2.05 | 1.43 | 2.53 | 3.77 | 6.39 | 5.14 |
| 87 | 6 | 264 | 28.2 | 55.59 | 51.83 | 53.12 | 88.85 | 70.10 | 80.31 | 8.55 | 6.53 | 7.90 | 0.17 | 2.22 | 1.33 | 8.55 | 6.14 | 7.78 |
| 88 | 6 | 224 | 27.3 | 57.88 | 65.68 | 64.21 | 27.14 | 332.19 | 3.45 | 2.54 | 3.24 | 2.68 | 2.26 | 2.86 | 2.68 | 1.16 | -1.51 | 0.16 |
| 89 | 6 | 229 | 26.3 | 72.01 | 77.37 | 78.70 | 26.71 | 11.74 | 13.49 | 6.42 | 6.42 | 5.86 | 5.74 | 6.28 | 5.70 | 2.89 | 1.31 | 1.37 |
| 90 | 6 | 234 | 28.0 | 73.22 | 76.22 | 69.76 | 25.43 | 265.71 | 33.06 | 0.46 | 1.66 | 0.36 | 0.41 | -0.12 | 0.30 | 0.20 | -1.66 | 0.19 |

**Table S2.** Histological pigmentation variables obtained for each scenario (Sc.), for each individual (Ind.). The weight (Weig.) and length (Len.) of each trout are also included the average of the three measurements. L, lightness; a*, red; b*, yellow. Chroma (C* = (a^2^ + b^2^)^1/2^) and hue (h* = arctan(b*/a*) × 57.29) values were calculated in this method. The subscripts indicate the measurement number. (*) In scenarios 1 and 5, only two measurements were taken, with the third measurement indicated by a dash (-).

| **Ind.** | **Sc.** | **Weight** | **Length** | **L** | **a*** | **b*** | **h*** | **C*** |
| --- | --- | --- | --- | --- | --- | --- | --- | --- |
| 1 | 1 | 263 | 28.5 | 61.22 | 4.72 | 3.62 | 33.90 | 6.10 |
| 2 | 1 | 270 | 28.0 | 56.44 | 0.62 | -0.21 | 186.86 | 1.62 |
| 3 | 1 | 268 | 28.5 | 59.53 | 1.57 | -0.29 | 349.13 | 1.59 |
| 4 | 1 | 236 | 27.8 | 48.82 | 2.89 | 4.20 | 53.15 | 5.12 |
| 5 | 1 | 273 | 28.5 | 36.95 | 1.05 | 5.24 | 71.13 | 5.47 |
| 6 | 1 | 270 | 28.5 | 44.79 | 3.70 | 9.69 | 69.02 | 10.38 |
| 7 | 1 | 250 | 28.5 | 61.60 | 2.83 | -1.62 | 329.87 | 3.26 |
| 8 | 1 | 242 | 27.2 | 54.56 | 1.84 | 4.54 | 67.16 | 4.90 |
| 9 | 1 | 242 | 27.4 | 56.00 | 3.58 | 2.65 | 33.79 | 4.65 |
| 10 | 1 | 271 | 29.5 | 50.88 | 2.99 | 3.76 | 44.18 | 4.98 |
| 11 | 1 | 280 | 28.3 | 59.81 | 4.43 | 8.73 | 62.13 | 9.79 |
| 12 | 1 | 261 | 28.0 | 56.72 | 1.97 | 2.60 | 46.75 | 3.77 |
| 13 | 1 | 240 | 27.7 | 51.27 | 2.35 | 2.24 | 208.92 | 4.22 |
| 14 | 1 | 278 | 29.0 | 57.70 | 3.48 | 0.15 | 183.14 | 3.49 |
| 15 | 1 | 234 | 27.5 | 55.73 | 3.97 | 0.33 | 180.61 | 4.11 |
| 16 | 2 | 362 | 31.6 | 63.98 | 1.48 | -0.37 | 220.43 | 2.81 |
| 17 | 2 | 330 | 31.5 | 62.22 | 2.34 | 0.58 | 134.22 | 2.47 |
| 18 | 2 | 191 | 25.5 | 72.11 | -0.36 | -1.30 | 230.21 | 1.42 |
| 19 | 2 | 300 | 30.0 | 70.36 | 0.15 | -3.03 | 272.78 | 3.04 |
| 20 | 2 | 274 | 29.5 | 62.58 | 3.88 | 0.20 | 110.18 | 4.17 |
| 21 | 2 | 345 | 30.5 | 59.00 | 0.21 | -1.46 | 260.51 | 1.82 |
| 22 | 2 | 303 | 29.5 | 64.74 | -0.38 | 3.69 | 96.23 | 4.14 |
| 23 | 2 | 317 | 30.0 | 61.76 | -0.31 | 2.13 | 89.08 | 2.28 |
| 24 | 2 | 341 | 31.5 | 67.08 | 1.97 | -3.93 | 297.63 | 4.40 |
| 25 | 2 | 268 | 29.6 | 67.80 | 0.75 | -2.72 | 267.91 | 3.05 |
| 26 | 2 | 419 | 32.5 | 54.70 | 0.45 | 2.95 | 80.72 | 3.02 |
| 27 | 2 | 303 | 30.0 | 70.08 | -0.26 | -0.07 | 178.49 | 1.11 |
| 28 | 2 | 345 | 31.4 | 54.53 | 1.72 | 3.40 | 62.81 | 3.84 |
| 29 | 2 | 283 | 29.4 | 63.34 | -0.33 | -0.57 | 234.16 | 0.97 |
| 30 | 2 | 271 | 28.3 | 65.48 | 1.12 | -3.49 | 288.53 | 3.71 |
| 31 | 3 | 314 | 30.0 | 53.13 | 1.94 | 8.93 | 75.91 | 9.27 |
| 32 | 3 | 272 | 28.6 | 60.59 | 3.80 | 2.47 | 146.28 | 5.47 |
| 33 | 3 | 270 | 29.0 | 62.06 | 3.17 | 6.51 | 63.82 | 7.25 |
| 34 | 3 | 247 | 27.5 | 65.99 | 1.97 | -2.47 | 308.53 | 3.16 |
| 35 | 3 | 258 | 28.0 | 49.84 | -1.11 | 3.81 | 106.18 | 4.08 |
| 36 | 3 | 263 | 28.0 | 52.58 | 3.07 | 3.41 | 43.74 | 4.67 |
| 37 | 3 | 248 | 27.5 | 55.05 | 2.78 | 1.26 | 26.08 | 3.13 |
| 38 | 3 | 205 | 27.5 | 61.57 | 1.45 | -2.81 | 198.68 | 3.59 |
| 39 | 3 | 297 | 29.5 | 62.24 | 2.15 | 6.23 | 71.28 | 6.64 |
| 40 | 3 | 293 | 30.0 | 55.36 | 0.87 | 10.30 | 84.99 | 10.34 |
| 41 | 3 | 315 | 29.6 | 66.28 | 2.61 | 3.93 | 53.97 | 4.86 |
| 42 | 3 | 287 | 30.2 | 64.88 | 2.17 | 1.93 | 41.20 | 2.95 |
| 43 | 3 | 236 | 27.0 | 62.44 | 2.10 | -1.55 | 224.17 | 3.17 |
| 44 | 3 | 255 | 28.5 | 62.82 | 0.93 | 0.60 | 149.40 | 1.49 |
| 45 | 3 | 261 | 28.0 | 55.08 | 1.68 | 4.65 | 70.58 | 4.95 |
| 46 | 4 | 387 | 32.5 | 61.32 | 3.03 | -0.40 | 233.04 | 4.60 |
| 47 | 4 | 302 | 30.4 | 69.24 | 0.12 | -0.65 | 311.44 | 0.96 |
| 48 | 4 | 323 | 30.3 | 53.62 | 1.93 | 3.05 | 56.61 | 3.64 |
| 49 | 4 | 335 | 31.4 | 68.57 | 1.52 | -0.63 | 224.40 | 1.72 |
| 50 | 4 | 347 | 31.0 | 66.63 | 1.06 | -1.47 | 294.15 | 1.85 |
| 51 | 4 | 293 | 29.7 | 59.25 | 0.48 | -3.77 | 280.11 | 4.04 |
| 52 | 4 | 318 | 30.8 | 55.42 | 4.60 | -6.38 | 305.28 | 7.88 |
| 53 | 4 | 363 | 32.2 | 58.06 | 2.51 | -4.78 | 295.24 | 5.51 |
| 54 | 4 | 308 | 30.2 | 60.23 | 3.54 | -3.39 | 316.41 | 4.91 |
| 55 | 4 | 366 | 32.3 | 58.85 | 3.88 | -2.43 | 331.45 | 4.69 |
| 56 | 4 | 319 | 30.3 | 62.72 | 0.24 | 0.29 | 70.28 | 0.73 |
| 57 | 4 | 228 | 27.7 | 54.58 | 2.15 | -1.88 | 321.17 | 2.89 |
| 58 | 4 | 290 | 30.5 | 53.80 | 5.89 | -4.92 | 320.05 | 7.68 |
| 59 | 4 | 317 | 30.1 | 65.67 | 0.52 | -3.81 | 278.28 | 3.87 |
| 60 | 4 | 303 | 29.8 | 77.03 | 4.58 | -3.27 | 324.41 | 5.64 |
| 61 | 5 | 259 | 28.4 | 71.79 | 2.20 | 0.03 | 186.03 | 3.67 |
| 62 | 5 | 293 | 29.0 | 66.69 | 3.27 | -0.69 | 348.48 | 3.40 |
| 63 | 5 | 244 | 27.3 | 69.62 | 4.56 | 0.91 | 190.17 | 4.73 |
| 64 | 5 | 316 | 31.0 | 65.43 | 4.63 | 3.05 | 30.01 | 5.77 |
| 65 | 5 | 274 | 29.0 | 73.50 | 5.81 | -5.10 | 318.87 | 7.75 |
| 66 | 5 | 257 | 28.5 | 68.69 | 0.90 | -2.93 | 282.44 | 3.13 |
| 67 | 5 | 260 | 29.3 | 70.96 | 3.53 | -1.55 | 336.24 | 3.89 |
| 68 | 5 | 244 | 28.0 | 70.39 | -0.10 | -1.60 | 268.58 | 1.61 |
| 69 | 5 | 214 | 26.7 | 61.89 | 2.40 | -0.64 | 224.27 | 4.41 |
| 70 | 5 | 257 | 28.7 | 65.59 | 2.65 | 0.20 | 182.98 | 2.88 |
| 71 | 5 | 251 | 28.5 | 61.88 | 3.18 | 2.92 | 139.22 | 6.74 |
| 72 | 5 | 299 | 30.0 | 51.72 | 0.72 | -0.29 | 162.27 | 2.49 |
| 73 | 5 | 328 | 30.7 | 55.85 | 3.28 | 3.01 | 41.91 | 4.47 |
| 74 | 5 | 287 | 28.7 | 59.62 | 2.44 | 3.46 | 52.38 | 4.33 |
| 75 | 5 | 288 | 29.0 | 58.53 | 1.48 | 4.39 | 73.96 | 4.67 |
| 76 | 6 | 205 | 26.5 | 63.47 | 6.71 | 3.55 | 145.45 | 9.24 |
| 77 | 6 | 238 | 26.0 | 75.04 | 5.17 | 1.87 | 20.68 | 5.69 |
| 78 | 6 | 227 | 26.5 | 73.94 | 5.42 | 0.38 | 245.67 | 5.88 |
| 79 | 6 | 205 | 26.5 | 59.93 | 3.90 | 5.58 | 54.55 | 6.95 |
| 80 | 6 | 266 | 28.5 | 64.69 | 2.40 | -2.29 | 211.41 | 3.65 |
| 81 | 6 | 253 | 27.3 | 43.49 | 2.35 | 2.25 | 33.89 | 3.62 |
| 82 | 6 | 223 | 26.6 | 55.42 | 1.46 | 0.97 | 146.94 | 2.00 |
| 83 | 6 | 267 | 29.0 | 75.45 | 3.64 | 0.66 | 247.22 | 4.58 |
| 84 | 6 | 301 | 29.0 | 61.34 | 1.13 | 5.10 | 74.36 | 5.32 |
| 85 | 6 | 248 | 28.6 | 67.88 | 2.68 | 2.47 | 40.15 | 4.08 |
| 86 | 6 | 250 | 28.5 | 64.82 | 2.00 | 5.10 | 67.55 | 5.53 |
| 87 | 6 | 264 | 28.2 | 53.51 | 1.24 | 7.49 | 79.75 | 7.66 |
| 88 | 6 | 224 | 27.3 | 62.59 | 2.60 | -0.06 | 120.93 | 2.82 |
| 89 | 6 | 229 | 26.3 | 76.03 | 5.91 | 1.86 | 17.31 | 6.23 |
| 90 | 6 | 234 | 28.0 | 73.07 | 0.20 | -0.42 | 108.07 | 0.83 |
